# Combined electric and magnetic field therapy for bone repair and regeneration: an investigation in a 3-mm and an augmented 17-mm tibia osteotomy model in sheep

**DOI:** 10.1186/s13018-023-03910-6

**Published:** 2023-06-24

**Authors:** Salim E. Darwiche, Anna Kaczmarek, Peter Schwarzenberg, Brendan J. Inglis, Beat Lechmann, Peter Kronen, Stephen J. Ferguson, Hannah Dailey, Brigitte von Rechenberg, Karina Klein

**Affiliations:** 1 Musculoskeletal Research Unit (MSRU), University of Zürich, Winterthurerstrasse 260, 8057 Zürich, Switzerland; 2 Center for Applied Biotechnology and Molecular Medicine (CABMM), University of Zürich, Zürich, Switzerland; 3 Lehigh University, Bethlehem, PA USA; 4 Johnson & Johnson Family of Companies, Solothurn, Switzerland; 5 Institute for Biomechanics, ETH Zürich, Zürich, Switzerland

**Keywords:** Fracture healing, Ovine models, Electromagnetic field therapy, Bone

## Abstract

**Background:**

Therapies using electromagnetic field technology show evidence of enhanced bone regeneration at the fracture site, potentially preventing delayed or nonunions.

**Methods:**

Combined electric and magnetic field (CEMF) treatment was evaluated in two standardized sheep tibia osteotomy models: a 3-mm non-critical size gap model and a 17-mm critical size defect model augmented with autologous bone grafts, both stabilized with locking compression plates. CEMF treatment was delivered across the fracture gap twice daily for 90 min, starting 4 days postoperatively (post-OP) until sacrifice (9 or 12 weeks post-OP, respectively). Control groups received no CEMF treatment. Bone healing was evaluated radiographically, morphometrically (micro-CT), biomechanically and histologically.

**Results:**

In the 3-mm gap model, the CEMF group (*n* = 6) exhibited higher callus mineral density compared to the Control group (*n* = 6), two-fold higher biomechanical torsional rigidity and a histologically more advanced callus maturity (no statistically significant differences). In the 17-mm graft model, differences between the Control (*n* = 6) and CEMF group (*n* = 6) were more pronounced. The CEMF group showed a radiologically more advanced callus, a higher callus volume (*p* = 0.003) and a 2.6 × higher biomechanical torsional rigidity (*p* = 0.024), combined with a histologically more advanced callus maturity and healing.

**Conclusions:**

This study showed that CEMF therapy notably enhanced bone healing resulting in better new bone structure, callus morphology and superior biomechanical properties. This technology could transform a standard inert orthopedic implant into an active device stimulating bone tissue for accelerated healing and regeneration.

**Supplementary Information:**

The online version contains supplementary material available at 10.1186/s13018-023-03910-6.

## Introduction

Delayed or nonunions remain at the forefront of outstanding challenges in fracture healing. The incidence can vary depending on the fractured bone location and other comorbidities, with associated significant economic and clinical impacts [1].

Therapies using electromagnetic field technology have shown evidence of enhanced bone growth and regeneration at the fracture site [2]. Magnetic fields used for bone applications ranged from 0.01 to 2 mT and produced electrical fields within bone tissue ranging from 1 to 100 mV/cm [3–5]. The electric fields reported are within the ranges described in naturally induced piezoelectricity within bone tissue in response to dynamic mechanical loading [6, 7]. The reported modalities and parameters of applied electromagnetic fields vary greatly, however, across reported studies. For example, the frequency of pulses usually ranges from 1 to 50 Hz, duration of treatments from minutes to hours and the window of treatment application from days to months [8–16].

**Fig. 1:**
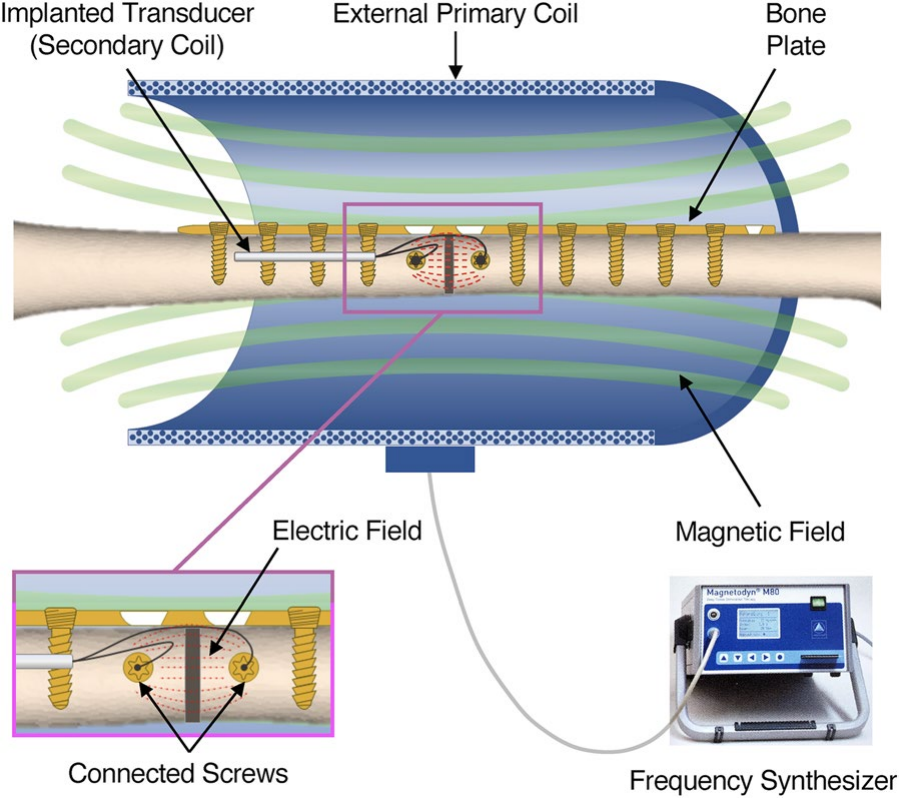
Schematic representation of the CEMF technology, showing the primary external coil, the magnetic field it generates when connected to the frequency synthesizer, the implanted transducer (secondary coil), which is connected to two screws placed on either side of the defect. The magnetic field generated by the primary coil induces an electrical field at the defect area, between the connected screws, without any physical transcutaneous connection to implanted components, thereby reducing infection risks

The technology tested in this study uses a combined electric and magnetic field (CEMF) and is therefore different from standard electromagnetic field devices: It uses a combination of magnetic and in situ amplified electric stimulation [17–19]. A primary external magnetic field activates an implanted transducer by electromagnetic induction, which generates an electric voltage between the connected screws (Fig. 1). The CEMF technology expands the functionality of already existing standard orthopedic and trauma implants to an on-demand, active stimulation device for bone healing. Indeed, the technology attaches to standard bone screws to deliver electromagnetic therapy and enhance bone healing by amplifying the electromagnetic stimulation locally at the fracture site.

The present in vivo study described herein was therefore designed to evaluate the impact of the CEMF technology on bone healing in two standardized sheep tibia osteotomy models, stabilized with internal fixation plates, based on previously established methods [20–23]. Specifically, in the first model, a 3-mm non-critical size defect was created, while the second model had a 17-mm critical size defect augmented with autologous bone grafts.

## Methods

### Animals and treatment groups

All animal experiments were conducted according to the Swiss laws of animal protection and welfare and were approved by the local governmental veterinary authorities (license no.: ZH183/17).

Fourteen adult, female, Swiss alpine sheep, averaging 28.7 months of age (24–30 months) and 66.98 kg body weight (62.70–76.60 kg) underwent surgery for the 3-mm gap model. Two animals had to be excluded, one due to implant failure of the internal fixation already 3 weeks after surgery and the other due to detachment of the implanted CEMF treatment device at some unidentified point during the postoperative in-life period (details in Additional file 1: Fig. S-1). The 17-mm graft model included 12 adult, female sheep averaging 31.2 months of age (24–34 months) and 71.49 kg body weight (58.60–86.65 kg). Animals were acclimatized for 7 days under test conditions and randomly assigned to two groups (CEMF control or CEMF treated) of six animals for each model (3 and 17 mm).

### Anesthesia and analgesia protocol

A pre-anesthetic examination was performed including general clinical examination, hematology and chemscreen.

After approximately 24 h of fasting and 30 min before induction of anesthesia, the animals were premedicated with buprenorphine (0.01 mg/kg BW im, Temgesic®, Reckitt Benckiser AG, Wallisellen, Switzerland, additionally administered 3 times after surgery (every 4–6 h)) and either xylazine (0.1 mg/kg BW im, Xylazin Streuli ad us. vet., Streuli Pharma AG, Uznach, Switzerland, animals 86.01–86.07) or medetomidine (0.005–0.01 mg/kg BW im, Medetor®, Virbac AG, Opfikon, Switzerland, animals 86.08–86.29). A booster for tetanus (3000 IU/sheep sc, Tetanus Serum Intervet, MSTD Animal Health GmbH, Lucerne, Switzerland) was administered subcutaneously. A catheter was placed into one jugular vein and prophylactic antibiotics (penicillin 30,000 IU/kg BW iv, Penicillin natrium Streuli ad us vet, Streuli Pharma AG, Uznach, Switzerland; gentamicin 4 mg/kg BW iv, Vetagent® ad us. vet., MSTD Animal Health GmbH, Lucerne, Switzerland), as well as a pre-emptive analgesic drug, carprofen (4 mg/kg BW iv, Rimadyl®, Zoetis Schweiz GmbH, Zurich, Switzerland), were given intravenously prior to surgery and 4 days thereafter. Anesthesia was induced with midazolam (0.1 mg/kg BW iv, Midazolam Sintetica, Sintetica AG, Mendrisio, Switzerland), ketamine (3–5 mg/kg BW iv, Ketanarkon® 100 ad us. vet., Streuli Pharma AG, Uznach, Switzerland) and propofol (0.6–2 mg/kg BW iv), the latter administered to effect.

After laryngeal desensitization with lidocaine spray, the trachea was intubated, and correct placement was confirmed by expired carbon dioxide monitoring (FetCO2). Anesthesia was maintained with a balanced anesthetic protocol, employing the administration of isoflurane (1–3%) in oxygen via an adult F-circuit, a variable rate infusion of propofol (1.88–3.82 mg/kg/h) and ketamine (1.04–2.22 mg/kg/h). The cornea was protected with ophthalmic ointment. Intraoperatively, Ringer’s lactate solution was administered at a rate of 5–10 mL/kg/h. Monitoring parameters included: electrocardiogram (ECG), heart rate, pulse rate and invasively or noninvasively measured blood pressures (systolic, mean and diastolic arterial) via an arterial catheter in an auricular artery. Furthermore, inspired and expired concentrations of carbon dioxide, oxygen and isoflurane as well as esophageal temperature and saturation of arterial blood (SpO_2_) were monitored. All parameters were constantly measured and recorded in 10-min intervals.

Additionally to the routine postoperative analgesia, paracetamol (10 mg/kg BW, iv) was given to the animals of the 17-mm graft model as deemed necessary, two/three times daily (BID-TID) every 8–12 h for 3–4 days, to mitigate potential pain from the autograft harvesting.

### Surgical procedure

The anesthetized sheep were placed in lateral recumbency with the upper, non-operated hindlimb in flexion and retracted craniodorsally. Operated hindlimbs were alternated from one animal to the next. A 16–18 cm incision was performed at the medial aspect of the tibia shaft of the hindlimb. The soft tissue and fascia were incised and dissected down to the bone.

Unless otherwise noted, all surgical instrumentation and implants mentioned were from DePuy Synthes.

**Fig. 2:**
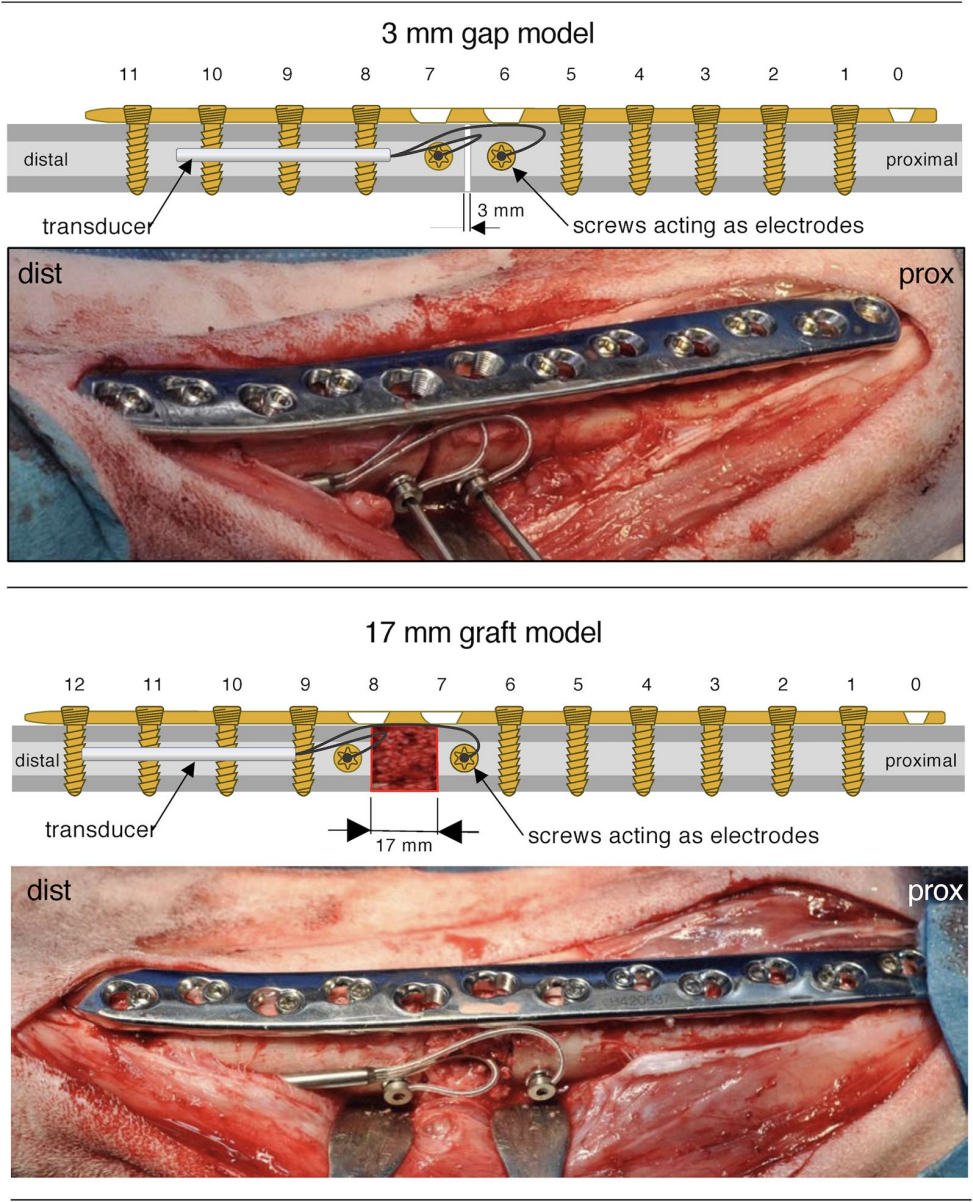
Schematic representation as well as representative pictures from surgery showing the fixation plates and screws as well as CEMF implants for both the 3-mm gap model and the 17-mm graft model. Note the orthogonal positioning of the CEMF screws in the antero-posterior axis, parallel to each other across the fracture gaps. The universal transducer is small enough to be placed under the musculature along the distal part of the tibia, and the wires connected to the CEMF screw heads without disrupting the fixation equipment

Fixation plates were contoured to the medial aspect of the tibia shaft. Custom designed cutting guides were temporarily fixed to the bone using Ø2.0 Kirschner wires at both ends and four monocortical screws (Ø2.7/3.5 mm, L20 mm, stainless steel), two proximal and two distal to the cutting area. An oscillating saw was used to perform the osteotomy through guiding slots under constant irrigation with saline. The cutting guide was removed, and the fragments were repositioned and fixed with a 12-hole or a 13-hole, broad, stainless steel, veterinary 3.5-mm locking compression plate using a 3-mm or 17-mm spacer, respectively. The already drilled holes were fixed again using the monocortical screws. The remaining screw positions were then drilled (2.8-mm drill bit and guide) and 3.5-mm bicortical screws in measured lengths were implanted. Step by step, the Kirschner wires then alternating monocortical screws were replaced by bicortical screws. Two plate holes were left empty at the defect area (Fig. 2). The 17-mm gaps were augmented using autograft harvested from the iliac crest of the same sheep. Non-augmented defects of this size with this kind of fixation would not spontaneously heal (Figure S-9).

After plate fixation, the CEMF technology was placed. Two parallel holes (either side of the gap, in the antero-posterior plane) were drilled bicortically (2.5 drill bit) using a customized drill guide, and the thread cut using a tapping device. Two 3.5-mm cortex screws were inserted, and the two wire caps of a Universal Transducer SI-ES (Neue Magnetodyn GmbH, Munich, Germany) were attached to the two screw heads (thereafter referred to as “CEMF screws”). The transducer was positioned under muscle tissue alongside the distal tibia (Fig. 2). Due to cap loosening in the first animals, redesigned transducer caps with tighter fixation were subsequently used (sheep 13 to 29). There were no transcutaneous CEMF components. Routine closure of wounds was performed.

### Postoperative radiographs, casts, CEMF therapy, fluorescent dye injection

Postoperative radiographs were taken in 3 different planes: antero-posterior (AP), mediolateral (ML), and in an angled plane between the AP and ML viewed from the cranial aspect of the operated limb. In addition to the postoperative Day 0 radiographs, radiographic follow-up was performed weekly starting at week 3 for the 3-mm gap model and every 3 weeks for the 17-mm graft model, until sacrifice. Radiographs were evaluated by two independent, blinded, board-certified reviewers (board-certified radiologist by the European College of Veterinary Diagnostic Imaging (ECVDI) and board-certified surgeon by the European College of Veterinary Surgeons (ECVS)), as previously described [24]. Semiquantitative radiographic union scores (0–30, high scores indicating more advanced healing) were given following a scoring system that captured callus formation and fracture line consolidation (Additional file 1: Table S-1).

All operated limbs were casted for the duration of the in-life period, with cast changes performed at each radiographic timepoint. The animals of the 3-mm gap model were kept in small groups, while animals of the 17-mm graft model were kept in suspension nets during the first 4 weeks after surgery. The health status (appetite, posture, alertness, respiration, weight bearing/gait, pain) of all animals was checked twice daily. Food and water were offered ad libitum. Antibiotics and analgesics were administered for 4 days post-OP (Additional file 1).

All sheep in the study were fitted with an outer CEMF coil (Neue Magnetodyn GmbH, Munich, Germany), custom shaped to fit a casted sheep hindlimb. CEMF treatment was applied for 90 min, twice per day, starting at day 4 after surgery until sacrifice after 9 or 12 weeks (Additional file 1: Fig. S-2). Sheep in the CEMF treated groups had their outer coils connected to the CEMF generators (Neue Magnetodyn GmbH, Munich, Germany). These were designed to produce an alternating sinusoidal magnetic field inside the outer coil (flux density of 5 mT, frequency of 2–20 Hz). The magnetic field induced 700 mV electrical potential within the implanted internal transducer thereby delivering an electromagnetic stimulation between the two CEMF screws acting as electrodes across the defect (Fig. 1, Additional file 1: Fig S-2).

Fluorescent dyes, which co-localize with newly deposited calcium, were injected to track new bone deposition and remodeling kinetics. Calcein green (5 mg/kg BW, sc) and Xylenol orange (90 mg/kg BW, sc) were injected at 3 and 6 weeks, respectively, post-surgery and Oxytetracycline (20 mg/kg BW, sc) injected 48-72 h prior to sacrifice.

### Sacrifice and macroscopic evaluation

At 9 weeks (3-mm gap) or 12 weeks (17-mm graft), sheep were sacrificed. The animals were stunned using a captive bolt and exsanguinated, resulting in death of the animal from cerebral anoxia. Operated and non-operated hindlimbs were harvested. The implantation sites and surrounding tissue were macroscopically examined, assessing the integrity of the implants, callus, fibrosis around the screw holes, metallosis and integration of the CEMF screws. Screws, CEMF implants and the plate were then removed.

### Micro-CT, morphometric analysis, and virtual torsional rigidity calculation

Micro-CT scans and analyses were performed using procedures described in our previous methodological validation studies [24]. All operated and non-operated tibiae from the CEMF and Control groups in both models were scanned using an XtremeCT II scanner (Scanco Medical AG, Wangen-Brüttisellen, Switzerland) with an X-ray voltage = 68 kVp, current = 1470 μA and scan isotropic resolution = 60.7 μm. A Scanco KP70 phantom (QRM, Moehrendorf, Germany) was also scanned to convert Hounsfield Units (HU) into mineral density values (mg HA/cm^3^).

A segmentation workflow was performed using the Mimics Innovation Suite (Materialise, Leuven, Belgium) to separate the new callus tissue from the existing cortical bone following a previously established procedure [24]. Morphometric analysis assessed callus volume (cm^3^), and callus mineral density was evaluated using a phantom calibration scan (mg HA/cm^3^). Virtual mechanical testing was also carried out on each animal. Scans were first down-sampled to 400 μm isotropic resolution, and then, quadratic tetrahedral finite element meshes were created in 3-Matic with maximum edge lengths of 0.4 mm on surfaces and 0.85 mm for volumes. Element-wise mechanical properties were interpolated from voxel image intensities using a previously published elastic modulus scaling law for ovine cortical bone [24] and a dual-zone material model for bone and callus [25]. In the operated limbs, elements comprising soft tissue with a radiodensity below 665 mg HA/cm^3^ were assigned a modulus of 50 MPa. The Poisson ratio for all elements was 0.3. Simulated torsion tests were used to calculate the virtual torsional rigidity (Nm^2^/deg) of operated and non-operated contralateral tibiae.

### Biomechanical testing

Nondestructive biomechanical torsion tests were performed on all operated and contralateral non-operated tibiae as previously described [24]. The distal and proximal ends of the tibiae were embedded in Beracryl pots, and a custom-made fixture was used to load the samples onto an Instron E10000 electro dynamic testing machine (Instron, Massachusetts, USA). An axial 5N pre-load was applied, followed by an internal rotation at 5°/min until a maximum of 10 Nm or 12° was reached. Rotation (in degrees) and torque (in Nm) were recorded at a sampling rate of 20 Hz. The torsional stiffness was then calculated as a linear regression of the loading curve between 6 and 10 Nm (MATLAB, version R2016b). The torsional rigidity (in Nm^2^/deg) was calculated for all operated and non-operated tibiae. The torsional rigidity of the operated tibiae was also normalized by those of the contralateral non-operated tibiae and reported as a percentage.

### Histology and histomorphometry: processing and evaluation

Trimmed diaphyses of all operated tibiae were fixed in 40% ethanol, serially dehydrated in increasing concentrations of ethanol (50% to 100%), followed by defatting in xylene and infiltration in liquid methylmethacrylate (MMA). MMA was left to polymerize at 4 °C, then at room temperature followed by a last step at 37 °C. Polymerized bone blocks were cut along the tibia axis, in the plane of the fixation screws adjacent to the defect (EXAKT1 Band System 300/301, Exakt Apparatebau GmbH & Co. KG, Norderstedt, Germany). Thick sections were ground down to 700–900 µm, microradiographed (Faxitron Ultrafocus 100, Faxitron Bioptics LLC) to corroborate the structure of calcified tissues, mounted on Acropal slides (Perspex GS Acrylglas Opal 1013, Wachendorf AG, Basel, Switzerland), then surface stained with toluidine blue.

Sections were imaged using a Leica Z6 APOA microscope equipped with a Leica DFC 420C camera (Glattbrugg, Switzerland) using standardized magnification (acquisition using Imagic IMS software, Imagic Bildverarbeitung AG, Opfikon, Switzerland). Global histomorphometry was performed by manually color-highlighting old bone, callus (new bone and autograft material within defect), cartilaginous tissue, and non-bone tissue (e.g., fibrous tissue, fat, bone marrow), using a standardized pixel-detecting tool from Adobe Photoshop Elements 2019 (Adobe Systems, San Jose, CA). A sectoral histomorphometric analysis was performed by color-highlighting callus formation at the cis cortex, endosteally and at the trans cortex. The colored images were analyzed using the Fiji image processing package (ImageJ, version 2.0) to quantify the colored pixels/tissue. The total pixels within the area of interest (excluding background) were set as 100% and the percentage of the different tissues was quantified.

Native ground sections were imaged using a fluorescent microscope and camera (Leica DM 6000B, Leica DFC 350 FX, Leica Microsystems CMS GmbH, Mannheim, Germany) to detect and descriptively evaluate fluorescent dye at various timepoints after surgery (3 weeks, 6 weeks and 2–3 days prior to sacrifice). A stitching function (Leica LAS-X standard software, Leica Microscopes) was used to capture the entire section.

Blocks were then trimmed down to the defect area and adjacent CEMF screw holes, and thin sections (5 µm) were stained with toluidine blue, van Kossa/McNeal and Hematoxylin/Eosin (HE). Thin sections were evaluated using a light microscope (Leica DMR system, camera DFC320), focusing on biocompatibility parameters surrounding the CEMF screws according to ISO10993/6 guidelines (Scoring scheme: Additional file 1: Table S-2). Defect healing was scored (bone resorption/formation, endochondral ossification, callus maturity and defect unity) using surface-stained ground sections and stained thin sections at three different regions: fracture ends and defect at trans cortex, fracture ends and defect at cis cortex and endosteal defect area. The evaluation was performed by two independent observers (Scoring scheme: Additional file 1: Table S-3).

### Statistical analysis

The CEMF treated and Control groups were compared in either model. The statistically significant threshold was set at *p* < 0.05. For CT morphometry, VTR and histomorphometry data, independent sample two-tailed Student’s t tests were performed, Levene’s test was used to assess equality of variance and normality was assessed with the Kolmogorov–Smirnov test. Effect sizes were reported for significant differences using Cohen’s *d*. As data sets for biomechanics as well as semiquantitative parameters (radiographic scores, histology scoring) did not all exhibit normal distributions, a nonparametric Mann–Whitney U test was used for these parameters (IBM SPSS statistics, version 28).

## Results

### Results from 3-mm gap model

None of the 12 animals included revealed clinical abnormalities. Animals tolerated the external coils, and the CEMF therapy was successfully applied in the CEMF group without complications. Minor issues with CEMF technology placement during surgery (transducer cap loosening, transducer malfunction) were successfully resolved, mostly intra-operatively. In one sheep (86.12, CEMF group), a second minor surgery was required at week 5 to reattach the radiographically detected loosened transducer cap. After redesign, no cap loosening occurred (details in Additional file 1).

**Fig. 3:**
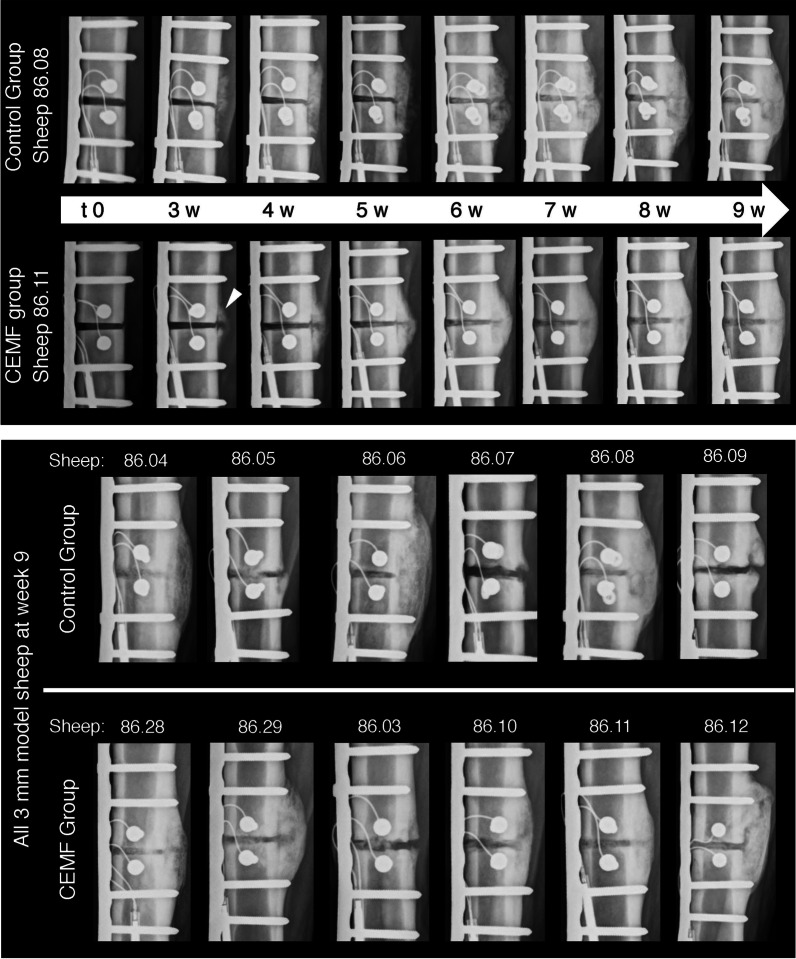
Representative radiographic follow-up in the 3-mm gap model from Control group sheep 86.08 and CEMF group sheep 86.11, starting at t0 (post-OP, day of surgery) to 9 weeks post-OP. The radiographs shown were taken in the antero-posterior plane. A periosteal callus formation at the trans cortex can already be seen at 3 weeks in the CEMF group sheep (white arrow), which is bridged by 9 weeks. Endpoint radiographs of all sheep at 9 weeks are shown in the bottom panel

Radiographic healing improved over time in all animals (Fig. 3, Additional file 1: Fig. S-3). Starting at 5 weeks, the CEMF group showed more advanced healing and systematically higher scores (about 2–3 points) compared to controls. Callus formation with CEMF was more advanced, smaller in size, smoother, more radio-dense and focused at the gap, compared to the Control (Fig. 3). At sacrifice (9 weeks), bridging callus was detected in 5/6 sheep in the CEMF and in 3/6 animals in the Control group, but the difference in total radiographic score was not statistically significant (Table 1).

**Table 1:** Semiquantitative and quantitative evaluations (radiographic scoring, CT morphometry, virtual torsional rigidity calculation, biomechanics, histomorphometry and histology scoring) performed on 3-mm gap model samples, showing means ± SD as well as *p* values comparing the CEMF and Control group

3-mm gap model				
Evaluation measure	Parameter	Control group (*n* = 6)	CEMF group (*n* = 6)	*p* Value
Radiographs	Scoring at 9 weeks [0–30]	15 ± 4	18 ± 4	0.180
CT morphometry	Callus density [mg HA/cm^3^]	659 ± 54	750 ± 87	0.053
	Callus volume [cm^3^]	11.0 ± 8.0	10.9 ± 4.1	0.977
Virtual torsional rigidity (VTR)	VTR OP tibia [Nm^2^/deg]	0.60 ± 0.42	1.00 ± 0.45	0.140
	VTR non-OP tibia [Nm^2^/deg]	1.01 ± 0.09	1.16 ± 0.19	0.139
	Normalized VTR vs. non-OP [%]	59 ± 39	84 ± 28	0.237
Biomechanics	OP Tibia Rigidity [Nm^2^/deg]	0.47 ± 0.39	0.95 ± 0.41	0.065
	Non-OP Tibia Rigidity [Nm^2^/deg]	0.88 ± 0.23	1.14 ± 0.29	0.124
	Normalized Rigidity vs. non-OP [%]	58 ± 46	81 ± 24	0.302
Global histomorphometry	Old bone [%]	27.3 ± 5.3	30.2 ± 6.4	0.407
	New bone [%]	23.0 ± 8.0	27.3 ± 7.1	0.354
	Non-bone [%]	49.7 ± 8.7	42.5 ± 7.2	0.151
Sectoral histomorphometry	Endosteal callus [%]	34.5 ± 18.5	21.5 ± 11.9	0.178
	Trans callus [%]	50.4 ± 21.1	59.9 ± 12.9	0.374
	Cis callus [%]	15.0 ± 8.3	18.6 ± 6.4	0.425
Histology (Defect healing score)	Bone resorption [0–4]	0.50 ± 0.62	0.27 ± 0.33	0.699
	Bone formation [0–4]	3.00 ± 1.19	3.61 ± 0.53	0.485
	Endochondral ossification [0–4]	0.89 ± 0.66	0.22 ± 0.40	0.093
	Callus maturity [0–4]	2.72 ± 1.25	3.50 ± 0.84	0.180
	Defect unity [0–4]	2.78 ± 1.17	3.56 ± 0.78	0.132

The score ranges for radiographic and histological scoring are indicated next to each parameter, and the detailed score schemes are shown in Additional file 1: Tables S-1 and S-3, respectively

* Indicates *p* < 0.05

The tissue adjacent to the implants did not show macroscopic abnormalities (e.g., hematoma, edema, encapsulation, granuloma). One locking head screw was broken at the neck in two control animals (screw 10 in 86.04, screw 9 in 86.05), and eight other minor locking head screw displacements were noted, without any directly associated macroscopic abnormalities. Metallosis at the screw head—plate junction was found in all sheep, particularly at the distal screw holes. All CEMF screws were firmly integrated in the bone with no macroscopic loosening and no metallosis. The implanted transducer and wires could be easily removed and were not encased in callus.

**Fig. 4:**
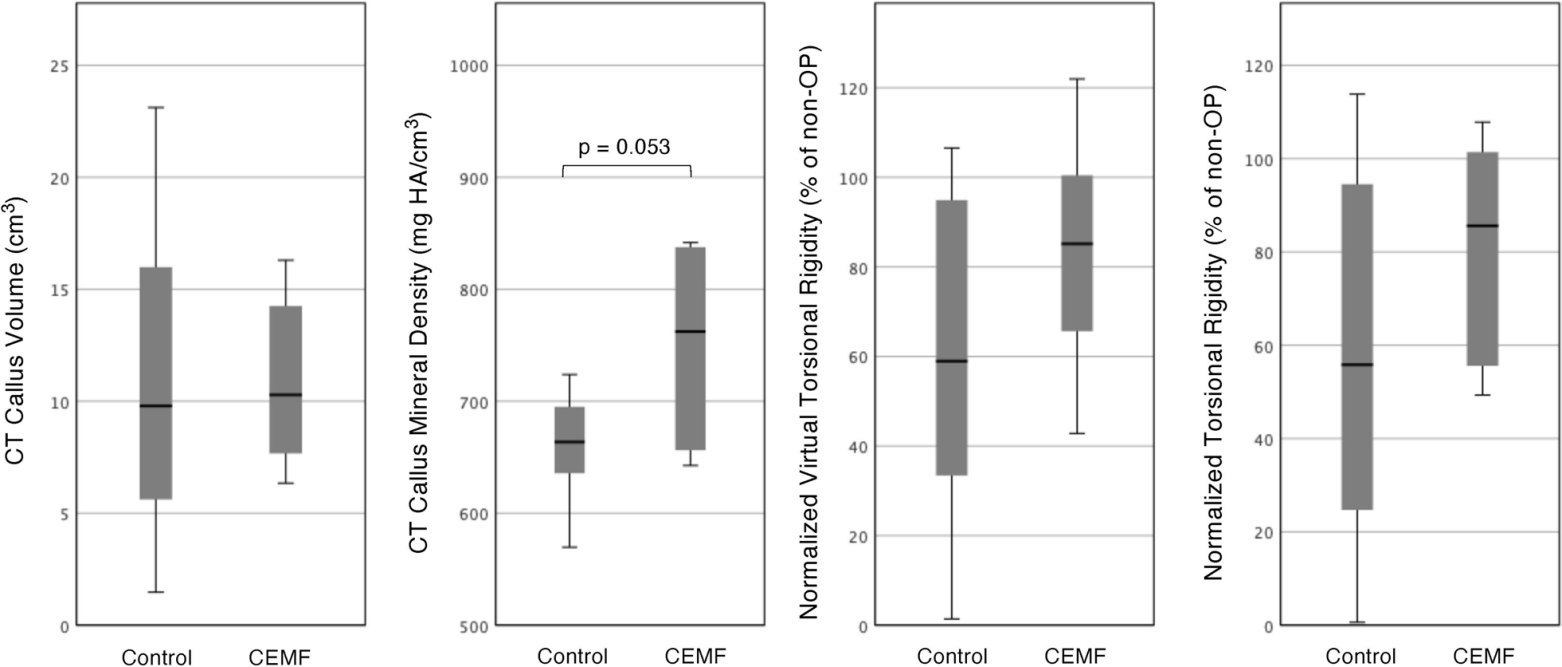
Two box plots to the left show Micro-CT morphometric analysis of callus volume and callus mineral density of operated tibiae in Control and CEMF groups of the 3-mm gap model. A denser callus average is seen in the CEMF group, but the difference between groups is not statistically significant (*p* = 0.053) due to variability. The two box plots to the right show normalized virtual torsional rigidity and normalized biomechanically measured torsional rigidity (shown as % of non-operated contralateral tibia rigidity) in Control and CEMF groups of the 3-mm gap model. A higher average virtual torsional rigidity and higher average biomechanical torsional rigidity is observed in the CEMF group, but the difference is not statistically significant due to variability.

A statistically detectable difference in callus volume in CT scans was not detected between the CEMF and Control group. Nevertheless, a higher mean callus density in the CEMF group was observed (*p* = 0.053; effect size *d* = 1.264) (Fig. 4, Table 1, Additional file 1: Fig. S-4).

The operated CEMF group tibiae had a higher mean virtual torsional rigidity than the operated Control group tibiae (Fig. 4, Table 1) (not statistically significant). Similarly, the mean biomechanically measured torsional rigidity of the operated tibiae was 2 × higher in the CEMF group compared to Control, but again not statistically significant (Fig. 4, Table 1). The biological variability coupled with the sample size did not allow the detection of a statistically significant difference between the groups, however.

**Fig. 5:**
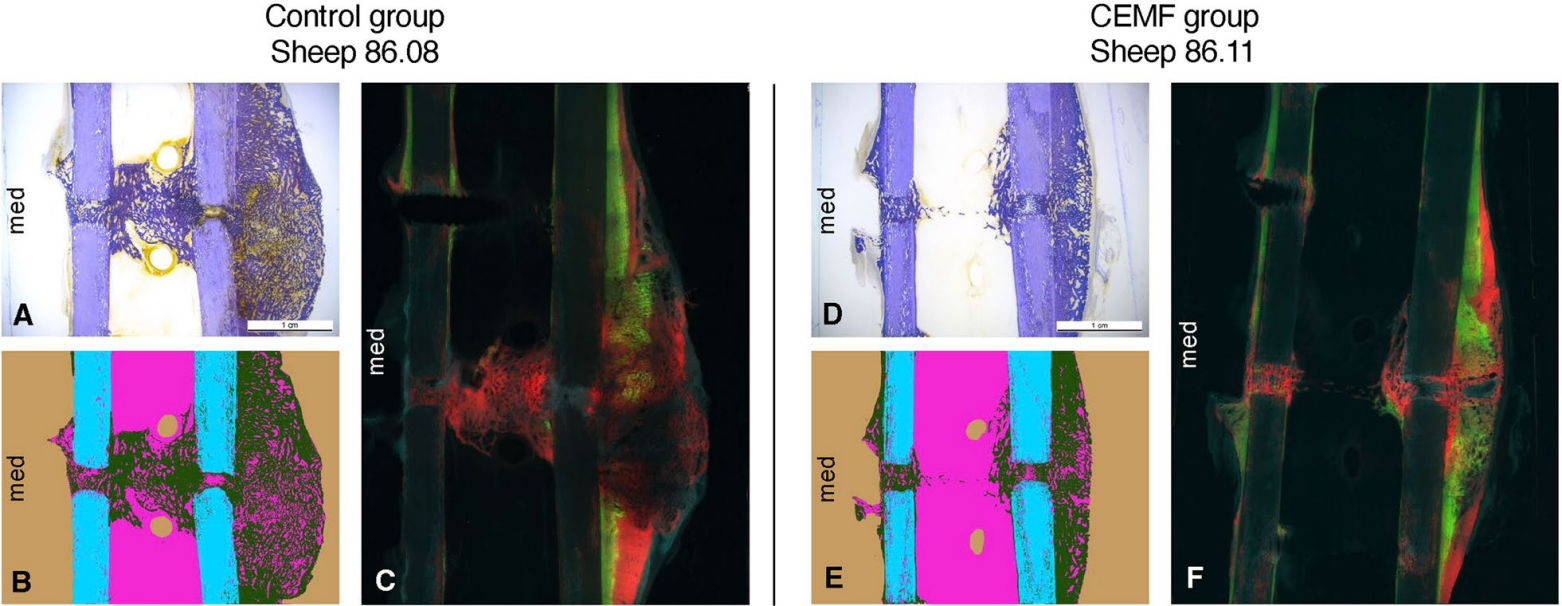
Representative histological, histomorphometric and fluorescent pictures of the 3-mm gap model from Control group sheep 86.08 and CEMF group sheep 86.11, showing a toluidine blue surface-stained ground sections (**A**, **D**), their respective histomorphometric segmentation (**B**, **E**) as well as the corresponding native section showing fluorescent dye deposition (**C**, **F**). The scale bar in ground section pictures A and D indicates 1 cm. The histomorphometric pictures (**B** and **E**) show background in beige, old bone in cyan, new bone in dark green and non-bone tissue in magenta. The fluorescent pictures (C and F) show overlays of Calcein green 3-week deposition in green, Xylenol orange 6-week deposition in red and Oxytetracycline 9-week deposition in blue. Overall, the interfragmentary healing and remodeling was more advanced in the CEMF group, associated with a smoother callus, versus a more irritated callus and less mature healing in the control sample. Toluidine blue surface-stained ground section pictures for all samples are shown in Additional file 1: Fig. S-5

Histomorphometry revealed comparable and good healing of the gap area in both groups. While no statistically significant differences were found, percentage of new bone was slightly higher in the CEMF group. The sectoral analysis of callus in the Control group showed a higher percentage of endosteal callus. The CEMF group showed a higher percentage of callus at the trans cortex, but the differences were not statistically significant (Fig. 5, Table 1, Additional file 1: Fig. S-5).

Fluorescent dyes indicating calcium deposition were mildly present at 3 weeks in both groups, mostly at the transcortical periosteal callus. At 6 weeks, a moderate deposition was visible in both groups endosteally and in the gap at both cortices, with a slightly more pronounced deposition in the CEMF group. Finally, the 9-week deposition pattern indicated an ongoing remodeling process, primarily in the cis and trans cortex of the CEMF group. The Control group showed more deposition endosteally, indicating a less advanced remodeling stage (Fig. 5).

**Fig. 6:**
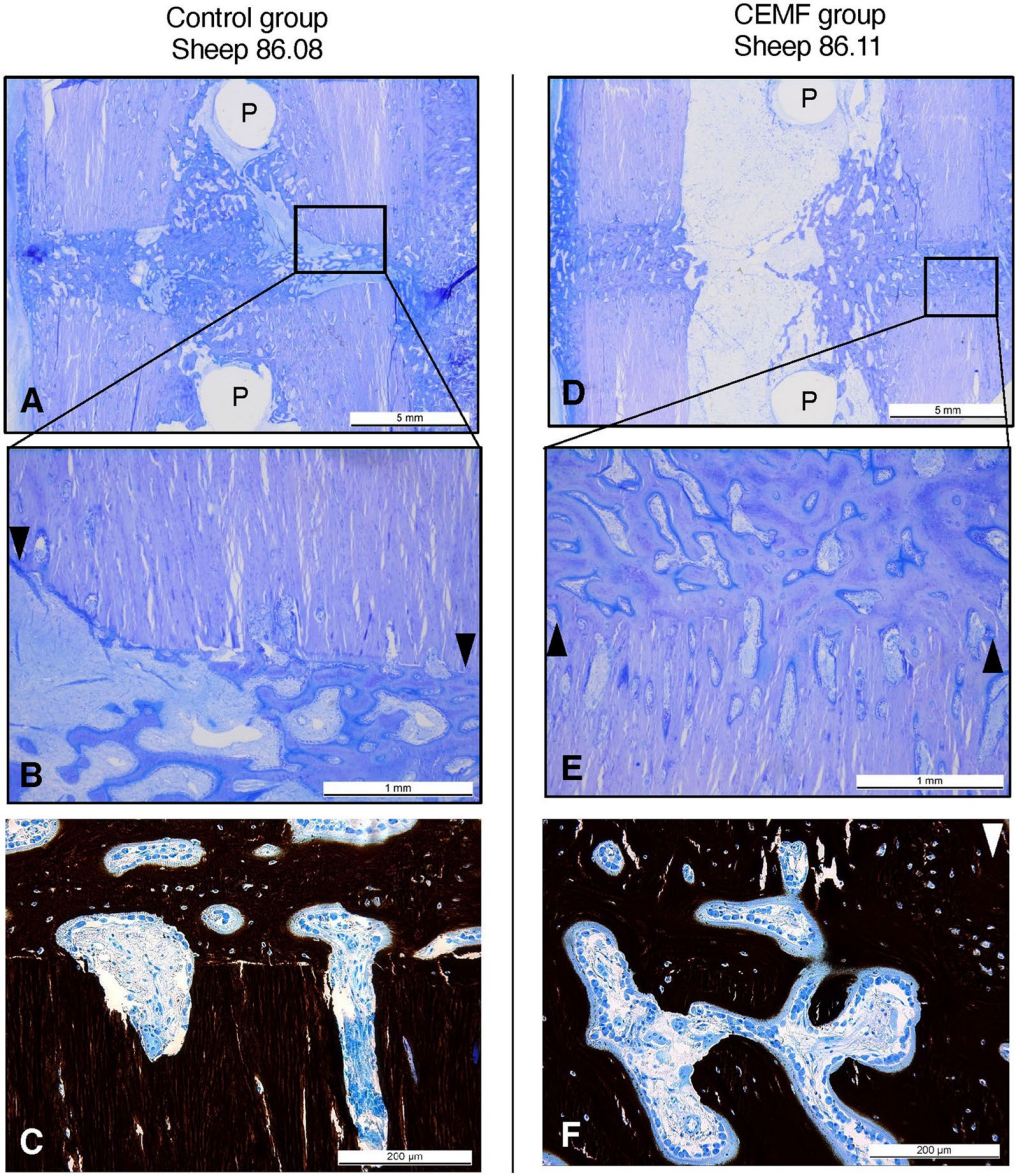
Representative histological pictures of the 3-mm gap model from Control group sheep 86.08 and CEMF group sheep 86.11, showing toluidine blue-stained thin sections at low (**A**, **D**) and high (**B**, **E**) magnification, with scale bars indicating 5 mm and 1 mm, respectively. CEMF screw holes are indicated with the letter “P.” Black arrows indicate the fracture line, specifically the transition between old and new bone, and point toward the fracture gap, showing an organized and integrated new bone formation at the fracture gap, particularly in the CEMF sample. Panels C and F show a von Kossa/McNeal staining of thin sections, highlighting the thicker osteoid seams observed in blue in the CEMF samples compared to the Control samples. Scale bars in panels C and F indicate 200 microns

Histologically, bone formation, callus maturity and defect unity scored higher in the CEMF group without a statistically significant difference (Table 1). The newly formed bone at the fracture ends (both cis and trans cortices) was already highly organized and integrated (Fig. 6), characterized by dense and regularly oriented structure in the longitudinal axis and a thicker osteoid seam with highly activated osteoblasts. Biocompatibility assessment did not reveal adverse effects.

### Results from 17-mm graft model

All animals of the 17-mm model showed good bone formation (minor complications: Additional file 1).

**Fig. 7:**
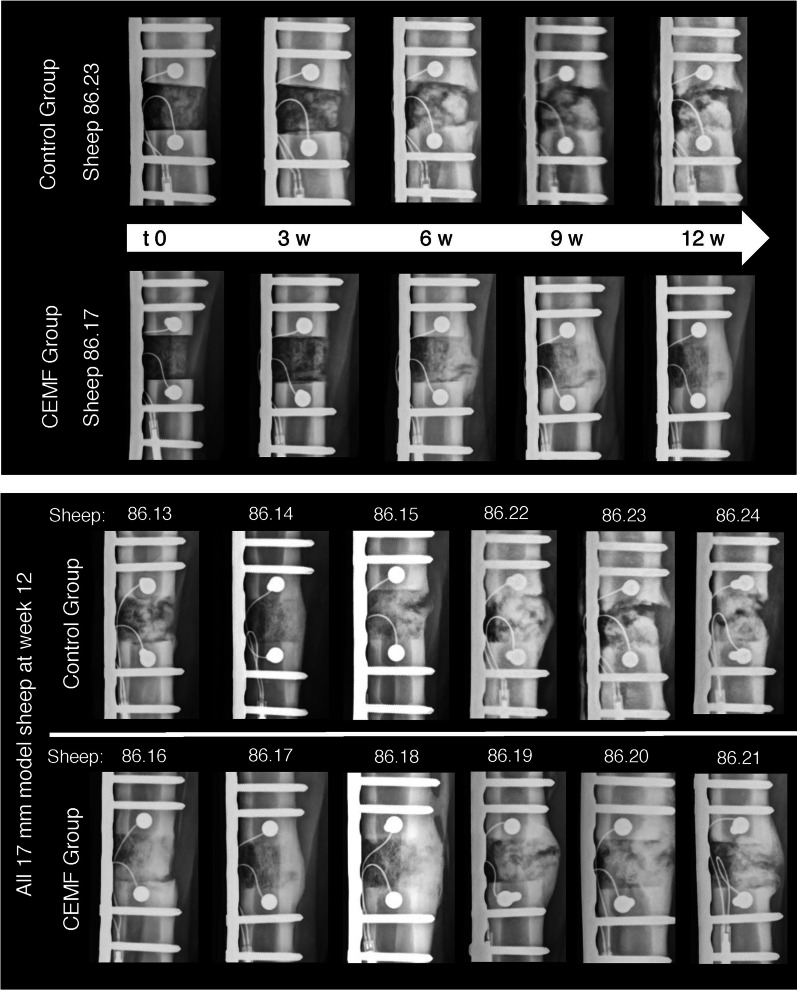
Representative radiographic follow-up in the 17-mm gap model from Control group sheep 86.23 and CEMF group sheep 86.17, starting at t0 (post-OP, day of surgery) to 12 weeks post-OP. The radiographs shown were taken in the antero-posterior plane. Autograft pieces are still visible in the Control group, with low-signal cartilagenous islets in the fracture gap, compared to the CEMF sample callus, which is more regular and throughout the defect, particularly focused on the trans cortex. Endpoint radiographs of all sheep at 12 weeks are shown in the bottom panel

While none of the defects were completely healed radiographically at 12 weeks, 5/6 CEMF sheep showed near-complete bridging of the defects, compared to only 1/6 Control sheep (Fig. 7). Differences were, however, not statistically significant (Table 2, Additional file 1: Fig. S-6). Overall, the defect filling in Control sheep appeared heterogeneous compared to the CEMF group. New bone formation around the autografts and increased callus formation within the defect were notable already at 3 weeks in the CEMF group and only at 6 weeks in the Control group (Fig. 7).

**Table 2:** Semiquantitative and quantitative evaluations (radiographic scoring, CT morphometry, virtual torsional rigidity calculation, biomechanics, histomorphometry and histology scoring) performed on 17-mm graft model samples, showing means ± SD as well as *p-*values comparing the CEMF and Control group

17-mm graft model				
Evaluation measure	Parameter	Control group (*n* = 6)	CEMF group (*n* = 6)	*p* Value
Radiographs	Scoring at 12 weeks [0–30]	15 ± 3	19 ± 2	0.093
CT morphometry	Callus density [mg HA/cm^3^]	674 ± 82	762 ± 56	0.055
	Callus volume [cm^3^]	7.3 ± 1.3	12.0 ± 2.3	0.003*
Virtual torsional rigidity (VTR)	VTR OP tibia [Nm^2^/deg]	0.32 ± 0.27	0.90 ± 0.33	0.007*
	VTR non-OP tibia [Nm^2^/deg]	1.10 ± 0.17	1.29 ± 0.21	0.115
	Normalized VTR vs. non-OP [%]	32 ± 28	69 ± 17	0.019*
Biomechanics	OP Tibia Rigidity [Nm^2^/deg]	0.32 ± 0.31	0.85 ± 0.37	0.024*
	Non-OP Tibia Rigidity [Nm^2^/deg]	1.10 ± 0.20	1.25 ± 0.21	0.256
	Normalized Rigidity vs. non-OP [%]	33 ± 34	67 ± 23	0.066
Global histomorphometry	Old bone [%]	17.3 ± 1.6	18.9 ± 6.5	0.574
	Callus [%]	31.4 ± 6.5	38.3 ± 7.3	0.115
	Cartilage [%]	3.9 ± 3.0	3.1 ± 4.2	0.691
	Non-bone [%]	47.4 ± 7.6	39.7 ± 7.5	0.111
Sectoral histomorphometry	Endosteal callus [%]	41.8 ± 7.9	35.9 ± 14.2	0.398
	Trans callus [%]	34.2 ± 8.1	44.5 ± 16.0	0.187
	Cis callus [%]	24.1 ± 6.5	19.6 ± 5.3	0.216
Histology (Defect healing score)	Bone resorption [0–4]	0.39 ± 0.65	0.00 ± 0.00	0.180
	Bone formation [0–4]	3.11 ± 0.72	3.89 ± 0.17	0.132
	Endochondral ossification [0–4]	1.50 ± 0.69	0.89 ± 0.34	0.180
	Callus maturity [0–4]	2.83 ± 0.69	3.67 ± 0.37	0.065
	Defect unity [0–4]	2.28 ± 0.83	3.56 ± 0.46	0.015*

The score ranges for radiographic and histological scoring are indicated next to each parameter, and the detailed score schemes are shown in Additional file 1: Tables S-1 and S-3, respectively

* Indicates *p* < 0.05

Tissues adjacent to the implants showed no alteration in their normal structure (e.g., hematoma, edema, encapsulation). The universal transducer and cables stayed in place did not interfere with the larger gap and could be easily removed. No severely displaced or broken locking head screws were detected. Metallosis at the screw head–plate junction was found in all sheep predominantly at the distal screw holes, but also at screw hole 6 in 7/12 animals. All CEMF screws were firmly integrated in the bone and no macroscopic loosening was observed.

**Fig. 8:**
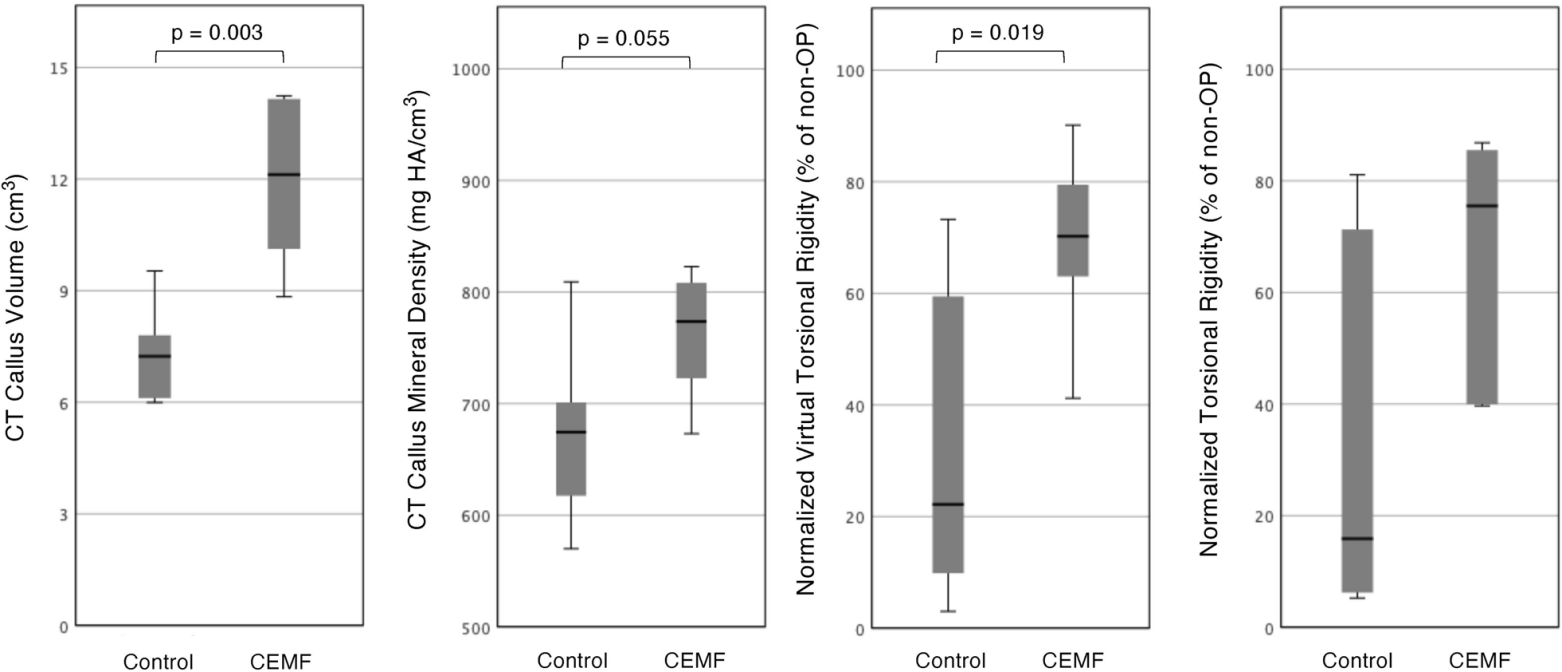
Two box plots to the left show Micro-CT morphometric analysis of callus volume and callus mineral density of operated tibiae in Control and CEMF groups of the 17-mm graft model. A significantly larger callus was seen in the CEMF group (*p* = 0.003), as well as a higher density average but that difference was not statistically significant (*p* = 0.055) due to variability. Two box plots to the right show normalized virtual torsional rigidity and normalized biomechanically measured torsional rigidity (shown as % of non-operated contralateral tibia rigidity) in Control and CEMF groups of the 17-mm graft model. A significantly higher virtual torsional rigidity was detected in the CEMF group (*p* = 0.019), and the biomechanically measured torsional rigidity in the CEMF group was more than 2.6 × that seen in the Control group

The callus volume in CT scans was significantly higher in the CEMF versus Control group (*p* = 0.003; effect size *d* = 2.46). While the callus density difference was not statistically significant, a higher mean density was seen in the CEMF group (*p* = 0.055) (Fig. 8, Table 2).

The operated tibiae in the CEMF group had a higher virtual torsional rigidity compared to the Control group (*p* = 0.007; effect size *d* = 1.93) (Fig. 8, Table 2).

The operated tibiae in the CEMF group showed distinctly superior biomechanical properties, with 2.6 × higher mean biomechanically measured torsional rigidity than the Control group (*p* = 0.024; effect size *d* = 1.54). The normalized operated limb rigidity relative to contralateral reached 67 ± 23% in the CEMF group versus only 33 ± 34% in the Control group (Fig. 8, Table 2).

**Fig. 9:**
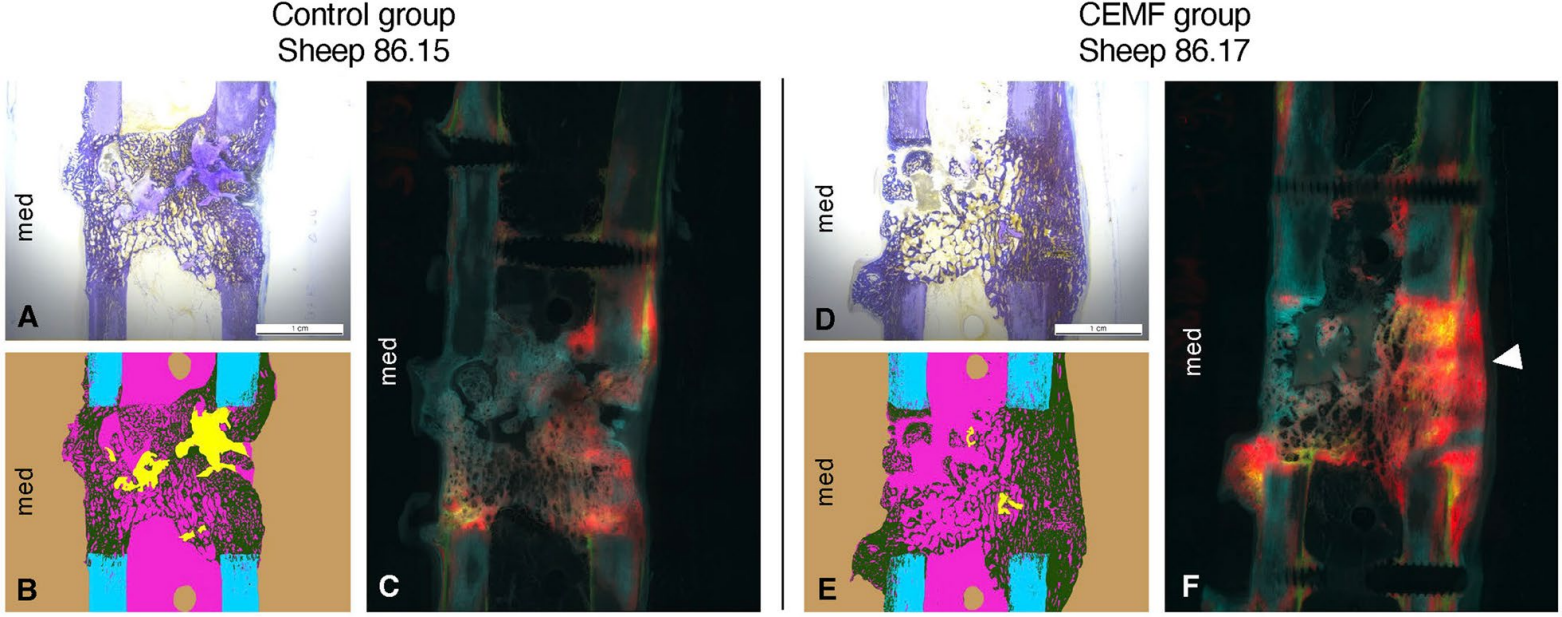
Representative histological, histomorphometric and fluorescent pictures of the 3-mm gap model from Control group sheep 86.15 and CEMF group sheep 86.17, showing a toluidine blue surface-stained ground sections (**A**, **D**), their respective histomorphometric segmentation (**B**, **E**) as well as the corresponding native section showing fluorescent dye deposition (**C**, **F**). The scale bar in ground section pictures A and D indicates 1 cm. The histomorphometric pictures (**B** and **E**) show background in beige, old bone in cyan, new bone in dark green, cartilaginous tissue in yellow and non-bone tissue in magenta. The fluorescent pictures (**C** and **F**) show overlays of Calcein green 3-week deposition in green, Xylenol orange 6-week deposition in red and Oxytetracycline 12-week deposition in blue. Deposition of bone at 6 weeks was more prominent at the trans cortex in the CEMF group (white arrow, panel **F**). Toluidine blue surface-stained ground section pictures for all samples are shown in Additional file 1: Fig. S-8

Histomorphometric analysis revealed good healing in both groups, with more advanced healing in the CEMF group. While differences were not statistically significant, a higher mean percentage of callus was detected in the CEMF group and a higher mean percentage of non-bone tissue in the Control group (Table 2). The sectoral analysis revealed the location of callus to be predominantly found endosteally in the Control group and predominantly at the trans cortex in the CEMF group (5–10% mean difference, not statistically significant) (Fig. 9, Table 2, Additional file 1: Fig. S-8).

Fluorescent dye deposition was slightly notable at 3 weeks, mostly within the defect area due to the remodeling process of the implanted autograft material. Compared to the 3-mm gap, the calcium deposition was not concentrated at the transcortical periosteum as it was removed during surgery in the 17-mm model. At 6 weeks, enhanced deposition was visible in a similar distribution across both groups. Intramedullary calcium deposition was the same in both groups. However, deposition at the fracture ends, especially at the trans cortex, was enhanced in 5/6 CEMF samples. At 12 weeks, the deposition was less visible compared to 6 weeks, indicating a more advanced but still ongoing remodeling process in both groups. Notably, some cortical areas in the fracture gap did not exhibit any activity. These areas corresponded with cartilaginous tissue (Fig. 9).

**Fig. 10:**
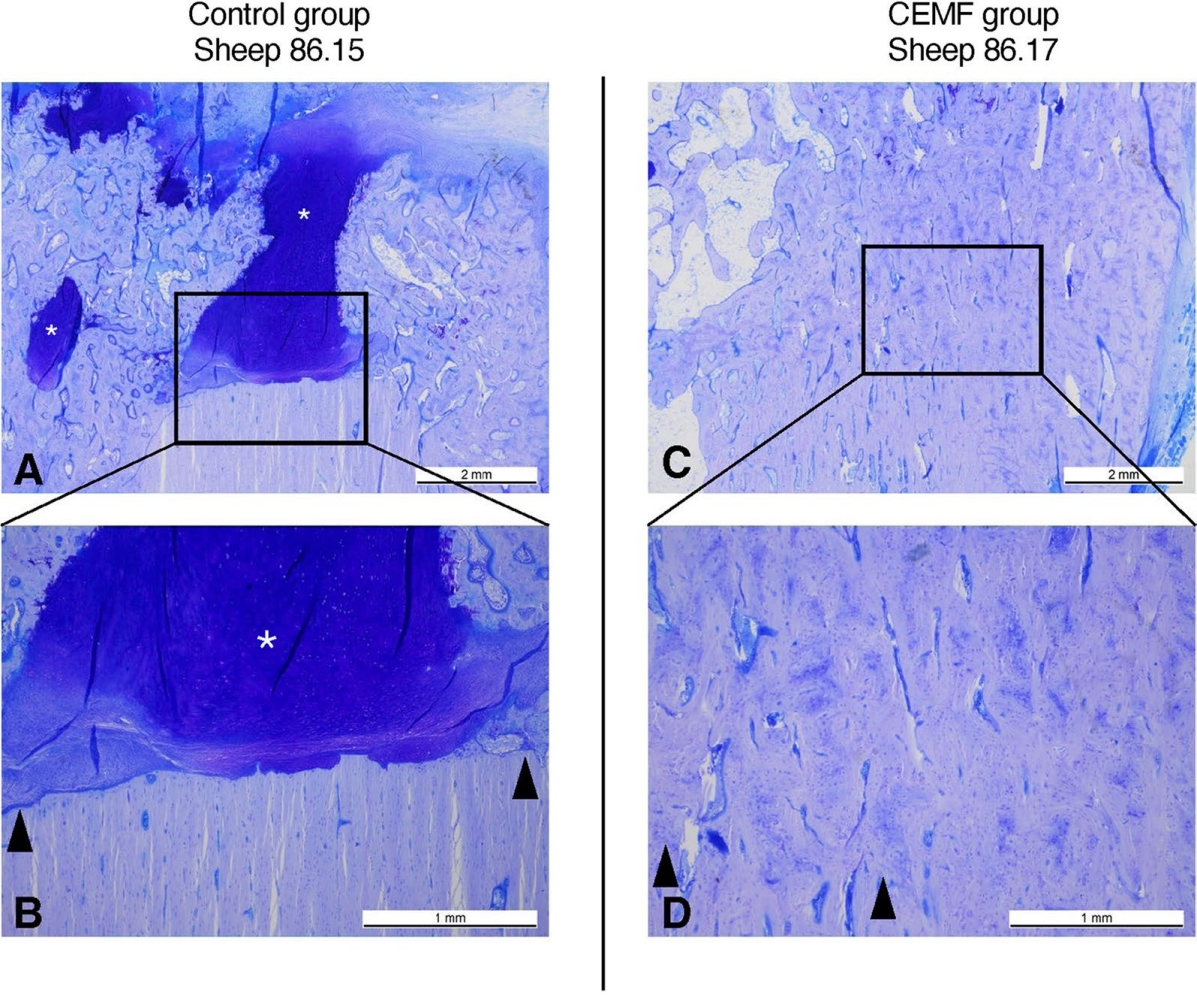
Representative histological pictures of the 17-mm graft model from Control group sheep 86.15 and CEMF group sheep 86.17, showing toluidine blue-stained thin sections at lower (**A**, **C**) and higher (**B**, **D**) magnification, with scale bars indicating 2 mm and 1 mm, respectively. Black arrows indicate the fracture line, specifically the transition between old and new bone, and point toward the fracture gap. In Panel D, the right most black arrow also indicates the trans cortex interface between cortical bone and callus. New bone formation in the fracture gap was more advanced in the CEMF group, as shown in C and D, versus the Control group, which showed notable cartilaginous tissue (dark purple staining glucosaminoglycan-rich tissue islets, shown with white asterisks) in the fracture gaps, indicating a less advanced healing stage

Assessment of biocompatibility revealed no abnormal reaction in the CEMF group compared to Control. The CEMF group scored better in all defect healing parameters compared to control, with statistically significant differences detected in defect unity (*p* = 0.015) (Table 2). At the fracture ends, new bone was moderately organized, characterized by dense, but still irregularly oriented structure in the CEMF group. In comparison, moderately organized but less dense new bone was found in the Control group, with notably more cartilaginous tissue islets as well as disorganized fibrous matrix (mixture of collagen fibers and proteoglycans) within the defect area and the fracture ends in most samples (Fig. 10).

## Discussion

This study demonstrated the effect of CEMF therapy on the acceleration and enhancement of the bone healing process overall in a standardized osteotomy model in sheep, resulting in more mature new bone structure, callus morphology and superior biomechanical properties. It was hypothesized, based on in vitro findings using the CEMF technology [26], that CEMF treatment would enhance bone healing in vivo. The CEMF technology showed a significant effect in the 17-mm at 12 weeks compared to the 3-mm model at 9 weeks, in which means may have been higher in the CEMF group, but higher variability in responses rendered the differences not statistically significant. Differences in healing kinetics, especially when assessed at 9 weeks, may not have been large enough or consistent enough in variability to detect in the 3-mm gap model, as the baseline ovine healing in this case was already rapid and robust. Nevertheless, higher mean values in biomechanics and morphometric density analyses, although not statistically significant, were seen also in the CEMF group of the 3-mm gap model, which was further supported by the histologically observed dense, well integrated, and regularly oriented new bone. In contrast, the timepoint at 12 weeks in the 17-mm defect group, which was expected to heal slower than the defects in the 3-mm gap model due to the size of the defect, was ideal to distinguish a clear effect of CEMF therapy showing a significantly larger callus volume (*p* = 0.003), higher virtual torsional rigidity (*p* = 0.007) and more than twice the torsional rigidity than control (*p* = 0.024).

The great advantage of the CEMF technology tested in this study is that it can be applied separately and independently from the standard fixation technique. The outer coil component can easily be placed when therapy is needed and, therefore, could be easily integrated into routine postoperative care. Indeed, the CEMF technology tested in this study could be applied in revision cases or after a delayed union has manifested. The CEMF technology could augment existing orthopedic tools with active stimulation, resulting in improved new bone structure, better callus morphology, superior biomechanical properties and thus, better fracture repair. The implanted transducer and wires can be permanent but can also be easily removed. The decision as to whether to keep the implants or undergo a second surgery to remove the implants would therefore likely be more related to the local health economics of a region than a surgical requirement. Future developments of the technology could also integrate the design in locking screws, for example, or other routinely used orthopedic fixation components to facilitate routine clinical use.

Some of the evaluation tools used in this study were translationally relevant (radiographs, CT scans, virtual torsional rigidity), while others cannot be translated to human clinical situations and can only be applied in preclinical animal studies (biomechanical tests, histology). Biomechanical testing can reveal important differences between healing outcomes at certain timepoints in the healing process. In secondary fracture healing in large mammals and humans, the rigidity of a healing long bone progresses through a well-known S-shaped curve that corresponds to the stages of mineralization and remodeling of the callus. Excluding nonunions, all fractures eventually reach equivalence with its intact state, although the timeline for this process can vary substantially based on the biological and mechanical conditions. A biomechanical test alone could detect for example a substantial difference in torsional rigidity between a fast healer and a slow healer at an earlier timepoint, but that difference would be difficult to detect at a later timepoint, even though the pathways of healing were different. In this situation, other analysis approaches such as histology become critically important for assessing the maturing of the bridging and progress of late-stage remodeling. This highlights the importance of a multi-disciplinary evaluation strategy, particularly in preclinical in vivo studies, in order to analyze all facets of bone healing and generate a holistic assessment of the effect of the new technology.

Despite positive preclinical outcomes from the described CEMF study, the question remains whether such active devices can be widely deployed in the current economic, regulatory and clinical landscape. Clinical evidence for the efficacy of existing electromagnetic field devices is mixed, making it difficult for new technologies to distinguish themselves from the field. The most recent meta-analyses indicate that electromagnetic field therapy may be effective in accelerating union in fresh fractures [27]. Some large-cohort studies indicate some benefit of electric stimulators for the reduction of nonunion rates [28] and the benefit of electromagnetic field therapy in early stages of healing, where it increases the union success rate [29]. The focus on binary union/nonunion as a primary efficacy outcome may in fact create a barrier to regulatory approval, as inconclusive clinical findings may not necessarily reflect a lack of efficacy of the devices and therapies tested, but rather an inability to measure the full continuum of treatment-related responses. There is therefore a real need to test electromagnetic field stimulation clinically in difficult fractures using modernized research tools, in order to translate the promising findings in ovine studies such as the one herein into promising clinical outcomes. While the gamut of methodologies employed in preclinical studies cannot all be used in human trials (e.g., postmortem biomechanics, histology), complementing existing clinical research assessment methods (radiographic assessment, CT morphometric analyses) with virtual structural analyses such as virtual torsional rigidity assessment could be very valuable to get quantitative measures of healing. Moving away from nonunion rate as a primary measure of efficacy may, therefore, not only decrease the cost of clinical trials, but also bring devices more efficiently through regulatory approval and widespread clinical use.

A limitation of this study was that the mechanism by which CEMF therapy may enhance bone healing was not investigated in this clinically oriented experiment. It may involve a spectrum of responses, as the therapy was applied starting at day 4 postoperatively and therefore bridged various stages of bone healing, from inflammation to remodeling [4]. In vitro tests performed on the same CEMF technology showed evidence of enhanced osteoblast proliferation and osteogenic protein expression [26], which merit future investigation in a large animal model. Another limitation was the sample size (*n* = 6), which, combined with the biological variability expected in sheep, made it difficult to detect statistical differences, particularly in the 3-mm gap model. Weighing animal welfare and ethics considerations, however, against the outcome of the study, the sample size was nevertheless sufficient to detect the effect of treatment in the 17-mm graft model. Another methodological limitation to note was that some semiquantitative scores were not designed to distinguish between various advanced stages of maturation in bone healing and exhibited a ceiling effect. Qualitative observations (increased alignment, observed density) were therefore reported to attempt to tease out those differences. Increasing the granularity of semiquantitative assessments in later remodeling phases was not in the scope of this study but could be investigated in the future in order to better understand the underlying biological responses at later stages of bone healing.

## Conclusions

Overall, the CEMF technology tested in this study notably enhanced bone healing, resulting in better new bone structure, callus morphology and superior biomechanical properties. This technology could transform a standard inert orthopedic implant into an active device stimulating bone tissue for accelerated healing and regeneration.

## Supplementary Information

**Additional file 1**. Supplementary Data including details on excluded animals, details on clinical findings and complications, the radiographic, biocompatibility and defect healing scoring schemes, pictures of the external CEMF coils and straps, graphs showing total radiographic scores for all 3mm and 17mm model sheep, 3D renderings of all operated tibiae in both models, toluidine blue stained ground sections from all sheep and radiographs from a small negative control group showing the 17-mm gap would not spontaneously heal without augmentation.

## Data Availability

The datasets generated and/or analyzed during the current study are not publicly available due to confidentiality requested by the study sponsor, but are archived at the corresponding author’s institution (both paper and electronic raw data as well as materials, e.g., histology blocks and slides) and are available from the corresponding author on reasonable request.

## Abbreviations

AP: Antero-posterior
CEMF: Combined electric and magnetic field
CT: Computed tomography
HE: Hematoxylin/eosin
ML: Mediolateral
MMA: Methylmethacrylate
Post-OP: Postoperatively
VTR: Virtual torsional rigidity
