# Supplementary material for: Combined electric and magnetic field therapy for bone repair and regeneration: an investigation in a 3-mm and an augmented 17-mm tibia osteotomy model in sheep
Source: J Orthop Surg Res. 2023 Jun 24;18:454. doi: 10.1186/s13018-023-03910-6 (PMC10290367; doi:10.1186/s13018-023-03910-6)
Supplement: Supplementary file 1 — Additional file 1. Supplementary Data including details on excluded animals, details on clinical findings and complications, the radiographic, biocompatibility and defect healing scoring schemes, pictures of the external CEMF coils and straps, graphs showing total radiographic scores for all 3mm and 17mm model sheep, 3D renderings of all operated tibiae in both models, toluidine blue stained ground sections from all sheep and radiographs from a small negative control group showing the 17-mm gap would not spontaneously heal without augmentation. [file 13018_2023_3910_MOESM1_ESM.pdf]

# Supplementary File

| Table of Content | Page |
|---|---|
| **Figure S-1**: Excluded animals | 2 |
| Clinical findings and complications | 3 |
| **Table S-1**: Radiographic score scale | 4 |
| **Figure S-2**: External CEMF coils and  straps | 5 |
| **Table S-2**: Biocompatibility scoring scheme | 6 |
| **Table S-3**: Defect healing scoring scheme | 7 |
| **Figure S-3**: Total radiographic scores from all 3 mm model sheep | 8 |
| **Figure S-4**: 3D renderings of all operated tibiae of the 3mm gap model | 9 |
| **Figure S-5**: Toluidine blue stained ground sections of all 3mm gap model sheep | 10 |
| **Figure S-6**: Total radiographic scores from all 17 mm model sheep | 11 |
| **Figure S-7**: 3D renderings of all operated tibiae of the 17 mm graft model | 12 |
| **Figure S-8**: Toluidine blue stained ground sections of all 17 mm graft model sheep | 13 |
| **Figure S-9**: Negative control group sheep (17 mm non-augmented defect) | 14 |

**Figure S-1**: Excluded Animals

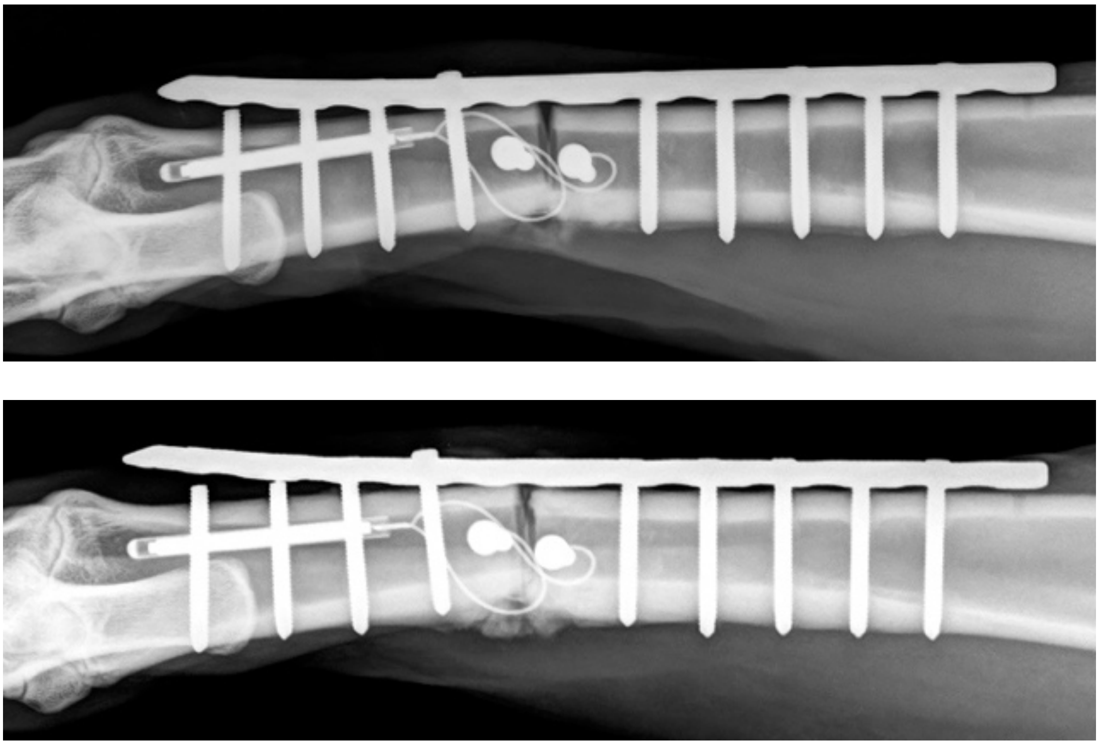

**Animal 86.01, top radiograph 3 weeks post-surgery, bottom radiograph 5 weeks post-surgery, collapse of fracture gap due to implant failure, after 3 weeks one broken screw visible, after 5 weeks two (11+12) and dislocation of screw 9.**

**Animal 86.01 (CEMF group, 3mm gap model)** had to be excluded from the group analysis due to implant failure of the internal fixation. A complete collapse of the fracture gap laterally at the trans cortex was already detected 3 weeks post-surgery during radiographic examination. The two most distal screws (hole 11 and 12) were broken and the screw in hole 9 (closest to the distal end of the defect) was displaced and bent). The sheep showed a very nervous, anxious and uncooperative behavior jumping a lot trying to escape. This was likely the primary cause of the fixation failure.

In **animal 86.02 (CEMF group, 3mm gap model)** the macroscopic evaluation at sacrifice at 9 weeks post-OP revealed a moderately detached transducer cap at the proximal Marvel screw with fibrous tissue between cap and screw head. As it was not detected with radiographic follow up, the exact time point of cap detachment could not be determined. Therefore, the CEMF treatment may not have been systematically administered throughout the in-life phase. This observation prompted the decision to exclude this animal from the analysis.

# Clinical findings and complications

In the 3mm gap model, in one sheep (86.10, CEMF group), the postoperative radiograph revealed a detached cap at the distal CEMF screw. This sheep, still under anesthesia, underwent a second surgery and the cap was fixed at the screw head again. Even though the anesthesia time was prolonged, no complications occurred during anesthesia nor postoperatively. Cap detachment was also detected radiographically 3 - 7 weeks post-surgery in 3 Control group sheep (86.04, 86.07, 86.08), but no reattachment was attempted as all 3 sheep were not receiving CEMF treatment. The proximal CEMF screw cap loosening in sheep 86.12 (CEMF group) was detected 5 weeks post-surgery. In this case, a second surgery was performed the same day to reattach the cap (5 cm long incision above proximal CEMF screw). Antibiotics (penicillin, gentamycin) and analgesics (Carprofen) were given intravenously for three days. No further complications evolved from this event and therefore this animal was included in the group analysis.

An anesthesia complication occurred with sheep 86.07 (control group). The animal developed a respiratory arrest immediately after extubation followed by pulmonary edema, which was immediately treated with furosemide. No further complications occurred during the recovery period and the in-life phase of this sheep. While appropriate actions were immediately taken to ensure survival of the sheep, return to full consciousness, sufficient spontaneous respiration and a stable circulatory function was achieved only within an hour.

The hypoxia and hypoperfusion of the region of interest during this time could have likely compromised the vitality and initial recruitment of cells needed during the early phase of bone healing. It is speculated that, from this complication, even later healing phases were affected. Indeed, at sacrifice, the fracture ends were movable and the gap unstable. The macroscopic and radiological findings in 86.07 were corroborated with all other evaluative methods, particularly with a low torsional stiffness (estimated at 0.07 Nm/deg, less than 1% that of the contralateral, non-operated tibia) and histological findings showing a delayed union.

In the 17mm graft model, one sheep (86.18, CEMF group) had a slight misalignment (ca. 1 mm) of the osteotomy cut, but the proximal and distal tibia alignment was good, nonetheless. Six weeks post-surgery, one animal (Control: 86.22) developed pressure sores at the udder caused by irritation and pressure due to the cast. The animal received antibiotics (amoxicillin, 15 mg/kg BW, im) and analgesics (carprofen 4mg/kg BW sc) for 8 days. After 10 days the animal fully recovered without further complications. Two animals (CEMF: 86.20, 86.21) lied down more than usual.

**Table S-1**: Radiographic Score Scale

| Radiographic Score Scale (range 0 - 30) | |
|---|---|
| **Cortical Callus Formation** | |
| **0** | no callus noted |
| **1** | callus not reaching into the defect |
| **2** | callus bridging the defect < 50% |
| **3** | callus bridging the defect >50% but <100 % |
| **4** | callus bridging the defect completely |
| **RUST Score** | |
| **1** | fracture with a fracture line and no callus formation |
| **2** | fracture with callus formation and a fracture line |
| **3** | fracture with bridging callus, but the fracture line is still visible across both cortices |
| **4** | complete bridging of the callus with no evidence of fracture line |
| **Callus Opacity** | |
| **0** | soft tissue opacity |
| **1** | < 50% of normal |
| **2** | > 50-100% of normal |
| **3** | > 100% (superimposition) |
| **Defect Opacity** | |
| **0** | soft tissue opacity |
| **1** | < 50% of normal |
| **2** | > 50-100% of normal |
| **3** | > 100% (superimposition) |
| **Cis-Cortex Callus Formation** | |
| **0** | no callus noted |
| **1** | callus not reaching into the defect |
| **2** | callus reaching into the defect, fracture line in callus visible |
| **3** | callus reaching into the defect, fracture line in callus less visible |
| **4** | callus reaching into the defect, fracture line in callus not visbile |
| **Trans-Cortex Callus Formation** | |
| **0** | no callus noted |
| **1** | callus not reaching into the defect |
| **2** | callus reaching into the defect, fracture line in callus visible |
| **3** | callus reaching into the defect, fracture line in callus less visible |
| **4** | callus reaching into the defect, fracture line in callus not visbile |
| **Cranial Cortical Gap** | |
| **0** | no callus noted |
| **1** | callus not reaching into the defect |
| **2** | callus reaching into the defect, fracture line in gap visible |
| **3** | callus reaching into the defect, fracture line in gap less visible |
| **4** | callus reaching into the defect, fracture line in gap not visbile |
| **Caudal Cortical Gap** | |
| **0** | no callus noted |
| **1** | callus not reaching into the defect |
| **2** | callus reaching into the defect, fracture line in gap visible |
| **3** | callus reaching into the defect, fracture line in gap less visible |
| **4** | callus reaching into the defect, fracture line in gap not visible |

**Figure S-2**: The external CEMF coils and straps.

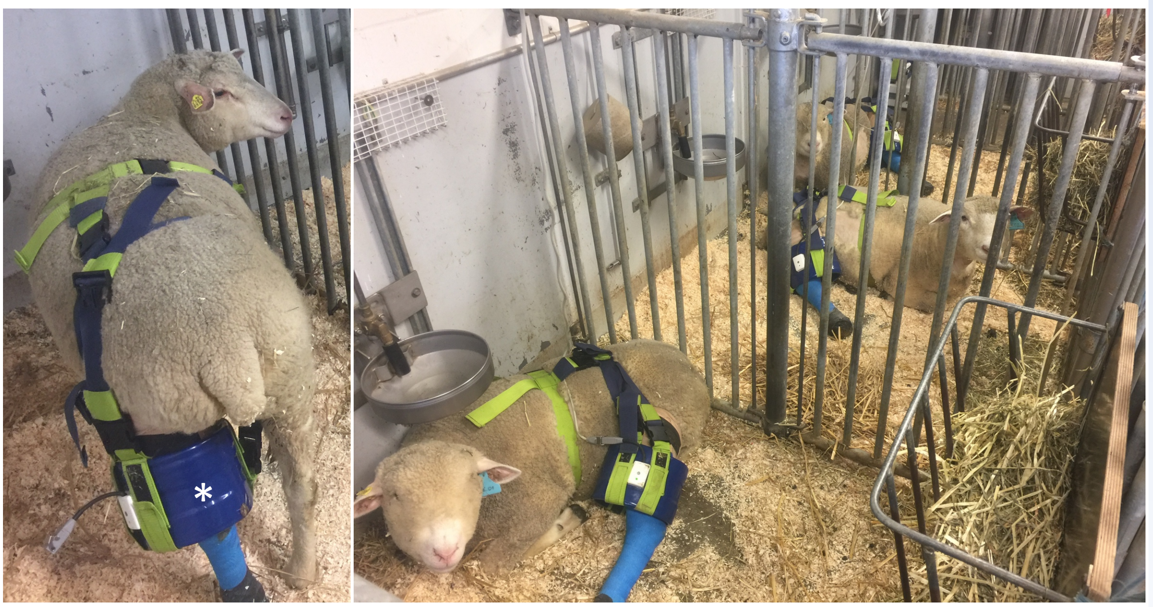

Sheep were fitted with an outer coil (white asterisk, left panel), custom shaped to fit a casted sheep hindlimb, for 90 mins, twice per day, starting day 4 after surgery until sacrifice. Custom straps were designed to allow ease of handling, ensuring sheep would be minimally disturbed without compromising the animal's ambulation and distributing the weight of the coil on the lower back of the sheep.

**Table S-2**: Biocompatibility Score Scheme
(CEMF screws and surrounding area)

| 1. Inflammation: Scoring scheme for inflammatory cells | | | | | |
|---|---|---|---|---|---|
| Cell type/response | Score | | | | |
| | 0 | 1 | 2 | 3 | 4 |
| Polymorphonuclear cells | 0 | rare, 1-5/phf[a] | 5-10/phf | severe infiltrate | packed |
| Eosinophils | 0 | | | | |
| Lymphocytes | 0 | | | | |
| Plasma cells | 0 | | | | |
| Macrophages | 0 | | | | |
| Giant cells | 0 | rare, 1-2/phf | 3-5/phf | | sheets |
| Necrosis | 0 | minimal | mild | moderate | severe |
| [a] phf per high-powered field (400x) | | | | | |

| 2. Tissue reaction: Scoring scheme for tissue reactions | | | | | |
|---|---|---|---|---|---|
| Response | Score | | | | |
| | 0 | 1 | 2 | 3 | 4 |
| Neovascularization | 0 | Minimal Capillary Proliferation, focal, 1-3 buds | Groups of 4-7 capillaries with supporting fibroblastic structures | Broad band of capillaries with supporting fibrotic structures | Extensive band of capillaries with supporting fibroblastic structures |
| Fibrosis | 0 | Narrow band | Moderately thick band | Thick band | Extensive band |
| Fatty infiltrate | 0 | Minimal amount of fat associated with fibrosis | Several layers of fat and fibrosis | Elongated and broad accumulation of fat cells about the implant side | Extensive fat completely surrounding the implant |

**Table S-3**: Defect Healing Score Scheme.

| **1. Bone activity** | | | | | |
|---|---|---|---|---|---|
| **Bone Resorption** | **Score** | | | | |
| | **0** | **1** | **2** | **3** | **4** |
| | no | 1-25% | 26-50% | 51-75% | 76-100% |
| **Bone formation** | **Score** | | | | |
| | **0** | **1** | **2** | **3** | **4** |
| | no, granulation tissue only | 1-25% | 26-50% | 51-75% | 76-100% |

| **2. Tissue character** | | | | | |
|---|---|---|---|---|---|
| **Endochondral ossification** | **Score** | | | | |
| | **0** | **1** | **2** | **3** | **4** |
| | no | 1-25% | 26-50% | 51-75% | 76-100% |
| **Maturity of callus/new bone** | **Score** | | | | |
| | **0** | **1** | **2** | **3** | **4** |
| | non union (fibrous tissue) | mainly mesenchymal tissue with some cartilage (endochondral ossification) | mainly cartilage and some new bone | more bone than cartilage | dense/mature bone |

| **3. Healing response** | | | | | |
|---|---|---|---|---|---|
| **Defect unity** | **Score** | | | | |
| | **0** | **1** | **2** | **3** | **4** |
| **Defect united in %** | 0 | not united, <25% | 26-50% united | 51-75% united | 76-100% united |

**Figure S-3**: Box plot showing total radiographic scores (range 0 – 30) from all 3 mm model sheep from week 3 to week 9.

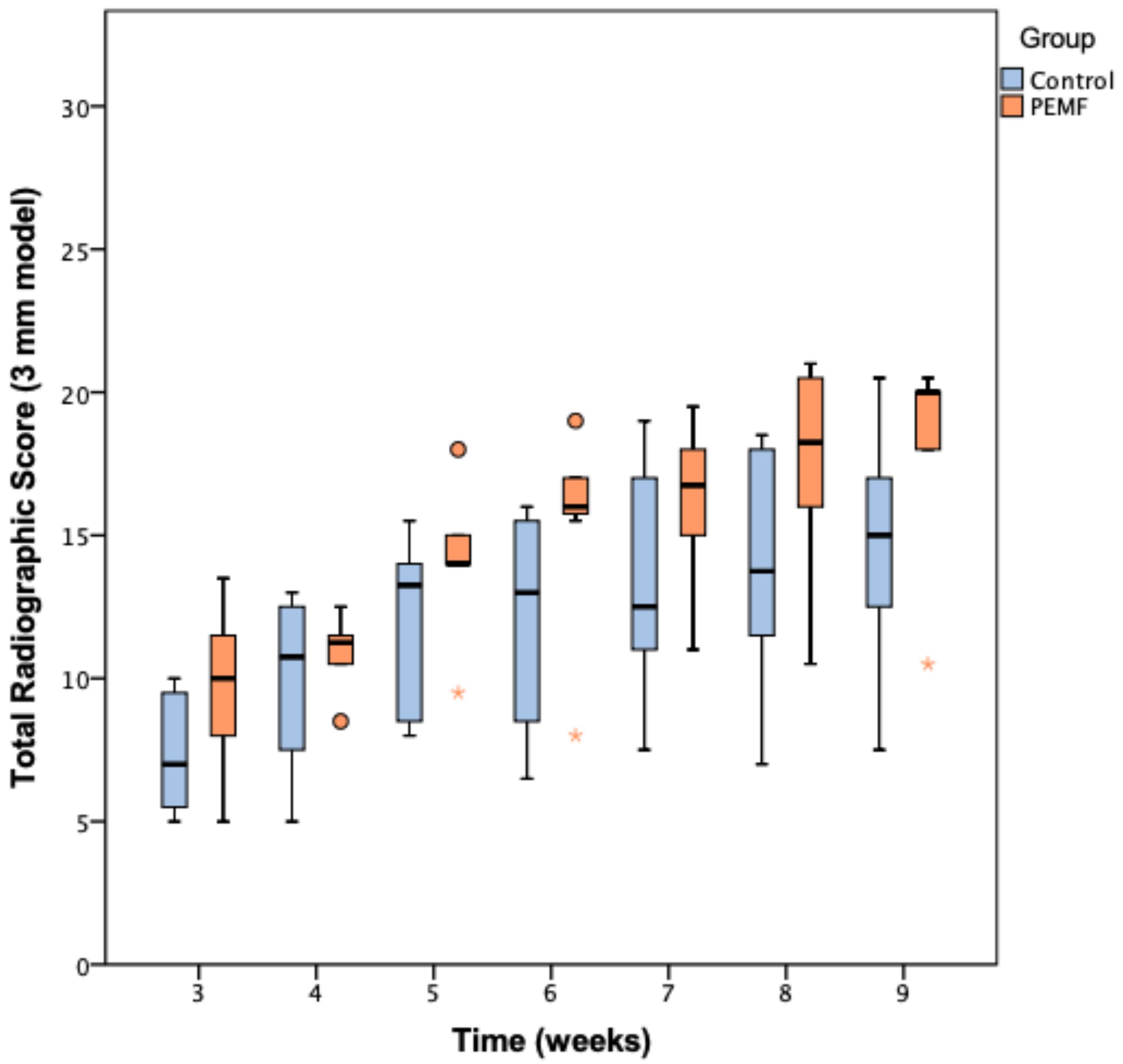

**Figure S-4**: 3D renderings of CT scanned operated tibia showing cortical bone (in yellow) and callus (in red) for all sheep of the 3 mm gap model.

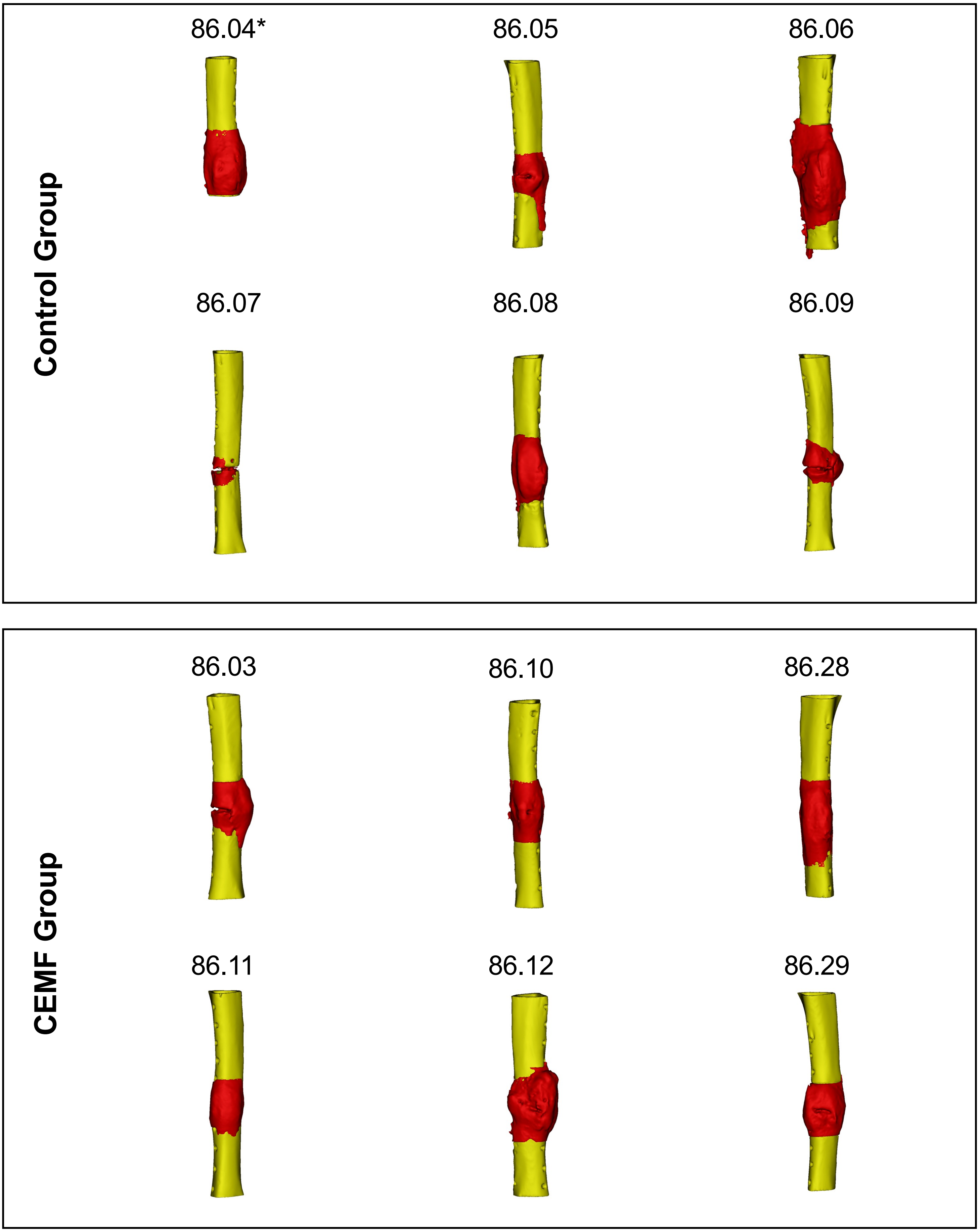


*Scan for 86.04 had a large image artifact from a retained screw fragment. Virtual model was cropped to remove the affected region.

**Figure S-5**: Toluidine blue surface stained ground sections for all sheep of the 3 mm gap model.

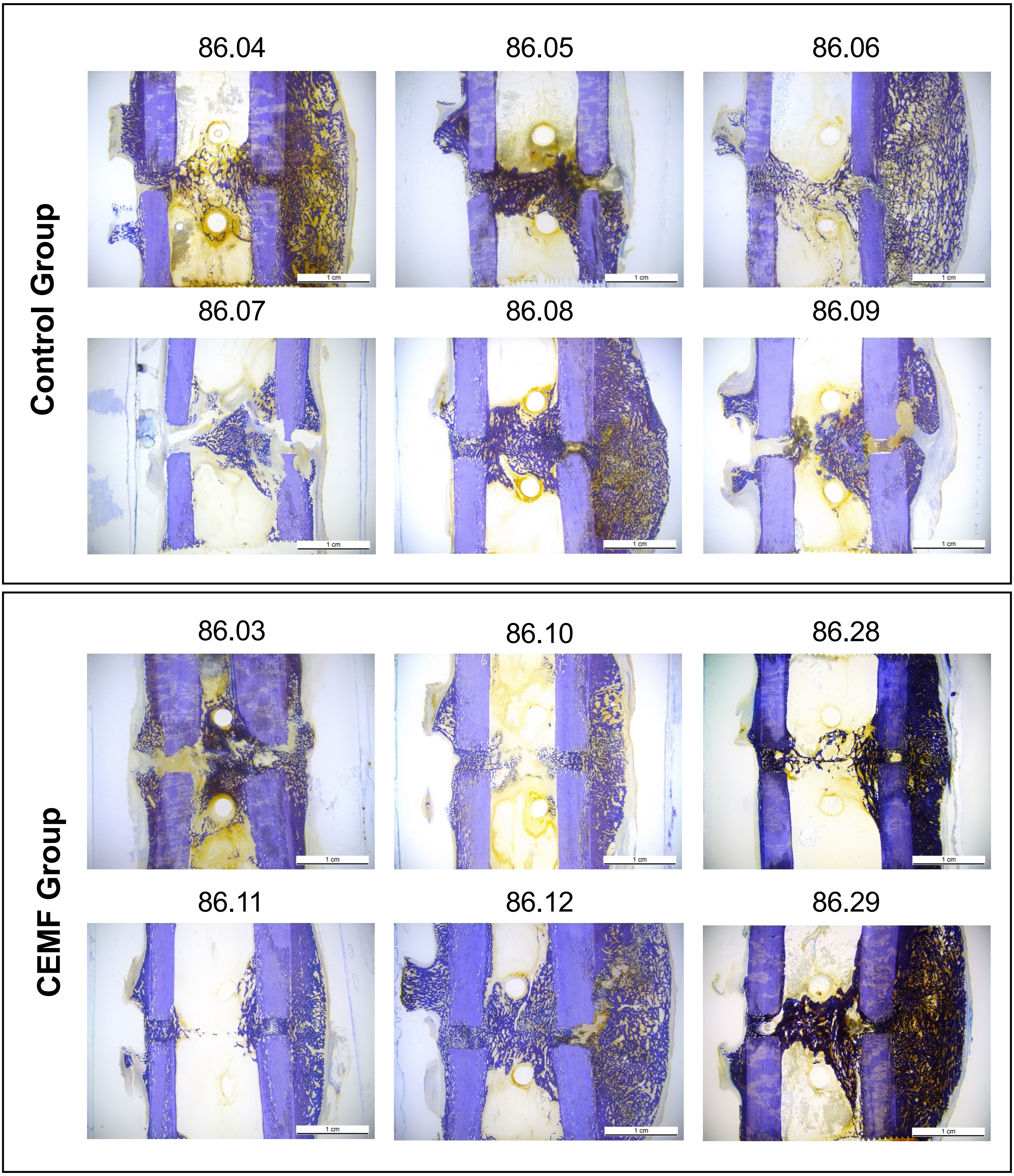

**Figure S-6**: Box plot showing total radiographic scores (range 0 – 30) from all 17 mm model sheep from week 3 to week 12.

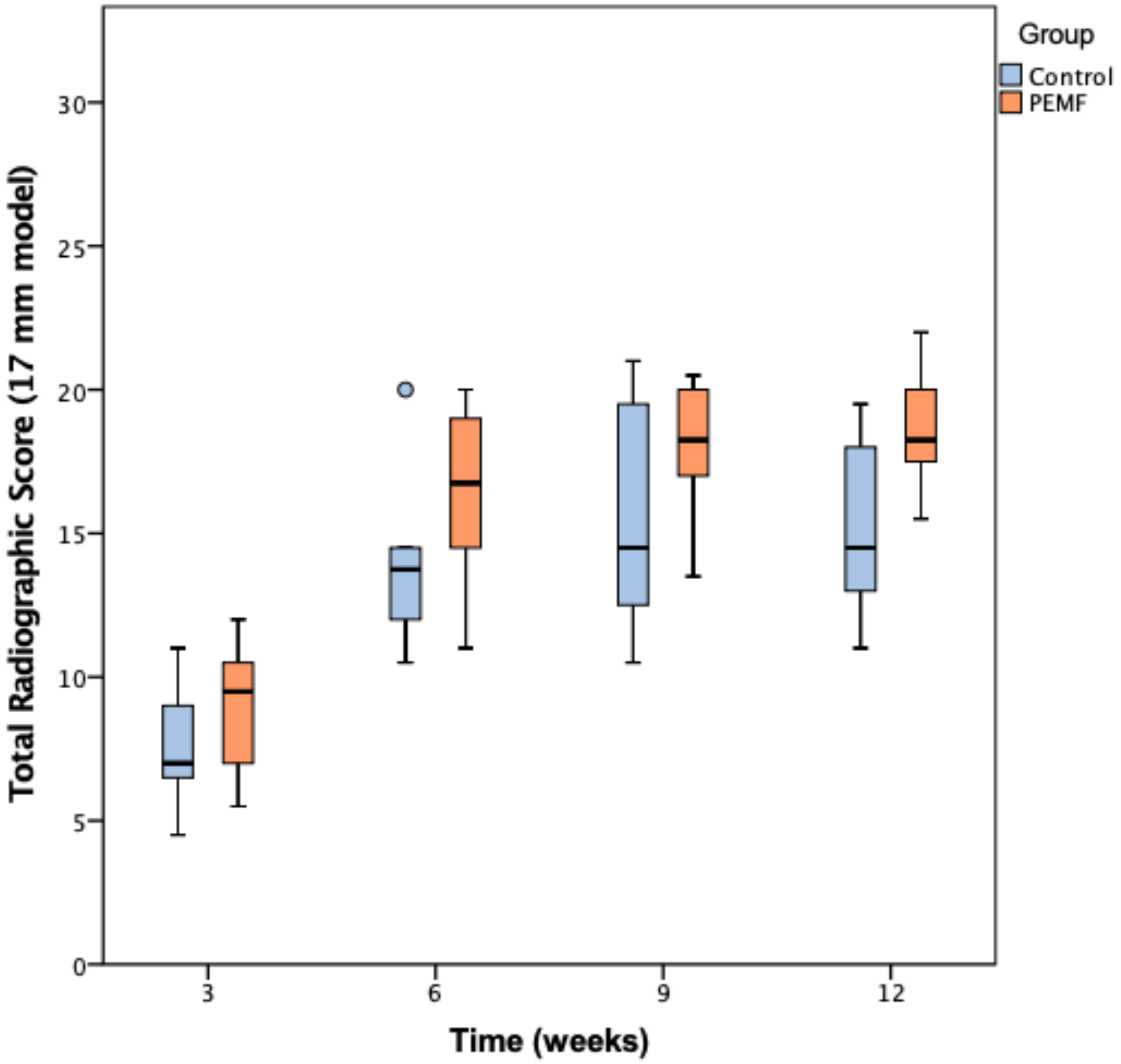

**Figure S-7**: 3D renderings of CT scanned operated tibia showing cortical bone (in yellow) and callus (in red) for all sheep of the 17 mm graft model.

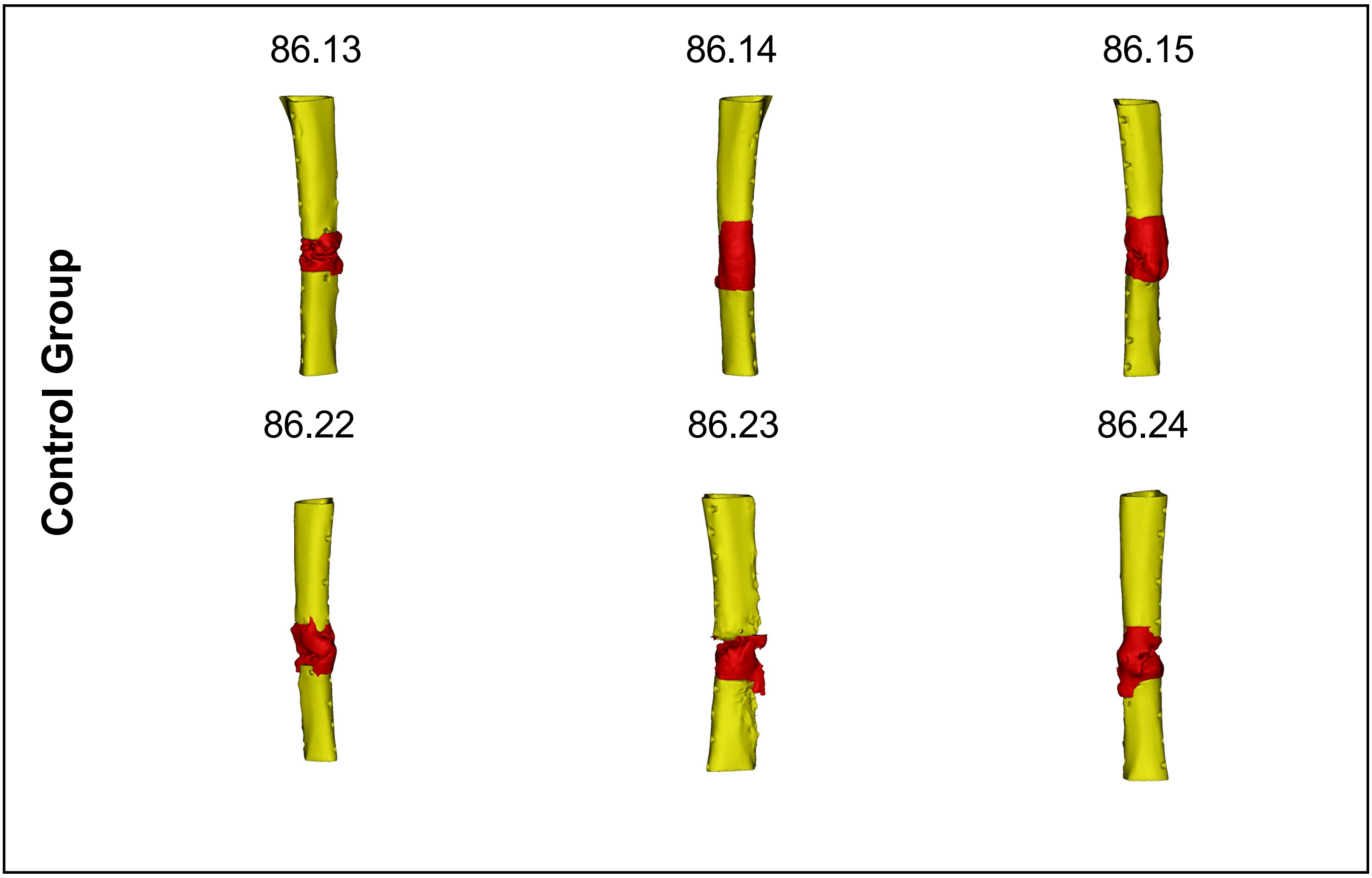


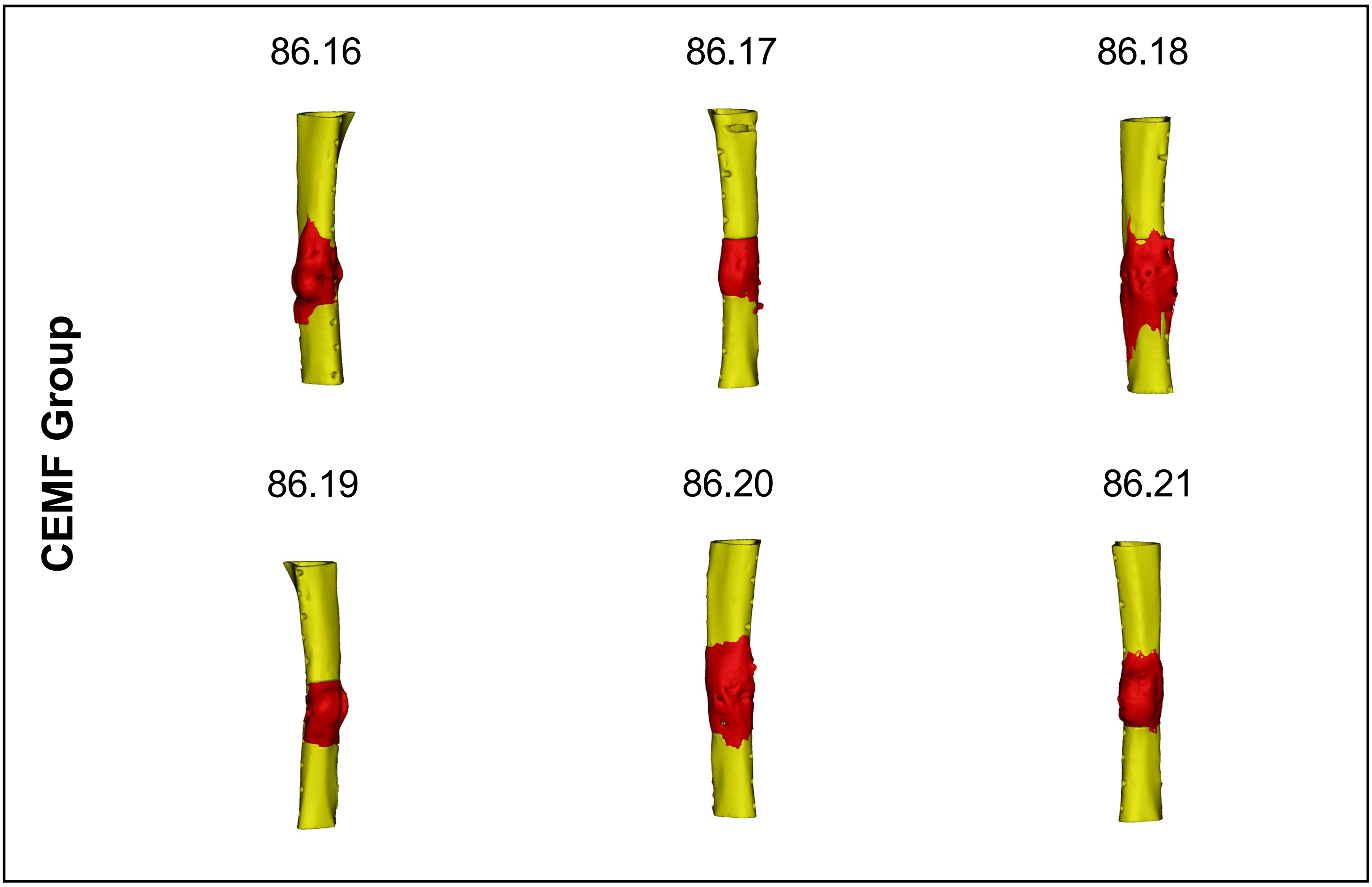

**Figure S-8**: Toluidine blue surface stained ground sections for all sheep of the 17 mm graft model.

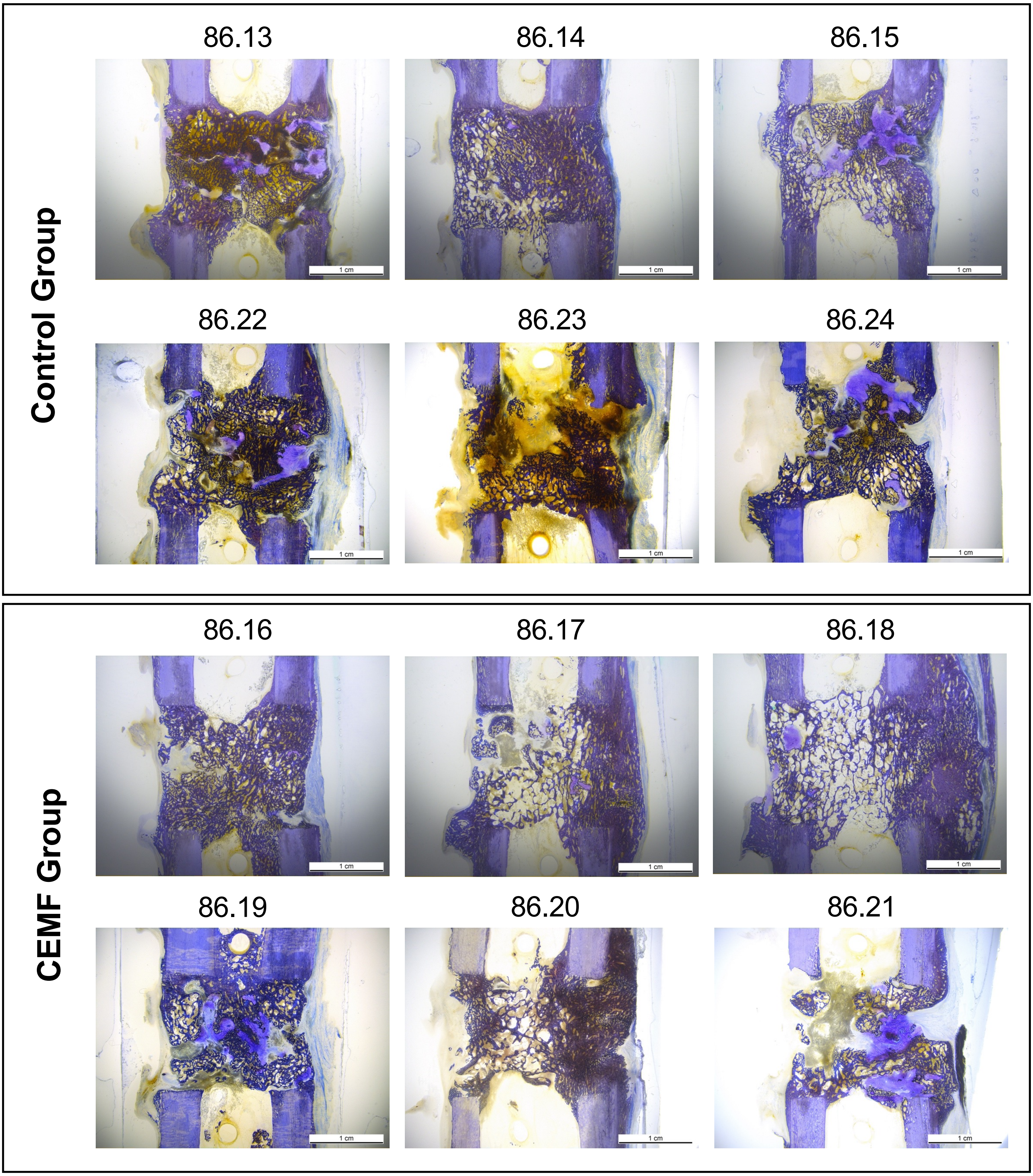

**Figure S-9**: Negative control group sheep (17 mm non-augmented defect).

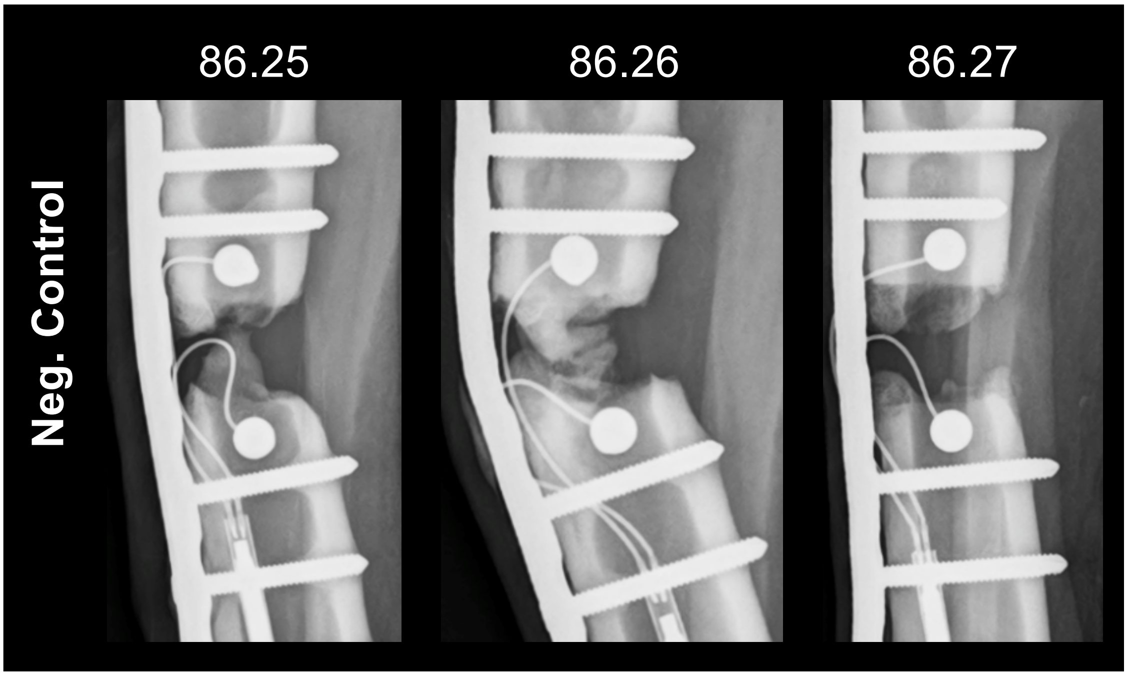


Negative control group sheep (17 mm non-augmented defect) Three sheep were operated as part of a negative control group. In this case, the tibia defect created was 17mm long and was not augmented with autologous bone. CEMF devices were implanted as with other CEMF or control animals, but CEMF treatment was not applied, as with Control group animals. All three sheep showed non-healed defects at 12-weeks post-operatively, as seen in the radiographs below. This finding showed that a non-augmented 17-mm gap tibia defect would not spontaneously heal in this model.
